# Intravenous administration of an engineered AAV9-gene-silencing vector suppresses human SOD1 and extends survival in an ALS mouse model

**DOI:** 10.1038/s41467-026-74169-8

**Published:** 2026-06-25

**Authors:** Fang Wan, Jinchen He, Hong Ma, Debora PiresFerreira, Veena Kumanan, Ji Sun Lee, Xiupeng Chen, Ran He, Qin Su, Thomas L. Gallagher, Sha Zhu, Gabriela Toro Cabrera, Lingzhi Zhao, Joan Shen, Alisha Gruntman, Robert H. Brown, Zuoshang Xu, Guangping Gao, Jun Xie

**Affiliations:** 1 Horae Gene Therapy Center, Department of Genetic and Cellular Medicine, University of Massachusetts Medical School, Worcester, MA USA; 2 Viral Vector Core, University of Massachusetts Chan Medical School, Worcester, MA USA; 3 Neushen Therapeutics US Inc., Burlington, MA USA; 4 Department of Neurology, University of Massachusetts Chan Medical School, Worcester, MA USA; 5 Department of Biochemistry & Molecular Biotechnology, University of Massachusetts Chan Medical School, Worcester, MA USA; 6 Department of Microbiology, University of Massachusetts Chan Medical School, Worcester, MA USA

**Keywords:** Amyotrophic lateral sclerosis, Gene therapy

## Abstract

Adeno-associated virus (AAV)-mediated gene silencing offers a promising strategy for achieving durable therapeutic effects with a single administration. Mutations in the human superoxide dismutase 1 *(*h*SOD1)* gene, inherited in an autosomal dominant manner, lead to motor neuron degeneration in amyotrophic lateral sclerosis (ALS)—a fatal neurodegenerative disease with no effective treatment. In this study, we employed AAV9 to deliver to the SOD1^G93A^ ALS mouse model artificial microRNAs targeting SOD1, embedded in dual miR-33 scaffolds driven by the promoter of the human survival motor neuron 1 (hSMN1) gene. A single intravenous injection achieved widespread and sustained suppression of SOD1, preserved α-motor neurons, maintained neuromuscular junctions (NMJs), and improved muscle function. These benefits are translated into significantly improved respiratory function, motor performance, and survival. Therapeutic efficacy was observed both when the treatment was administered pre-symptomatically and during symptomatic stages. Compared with previous AAV-based interventions, the survival benefit achieved in this IV delivery approach is unprecedented, supporting its potential for clinical translation in SOD1-linked ALS and other central nervous system (CNS) diseases caused by gain-of-toxicity gene mutations.

## Abstract (web summary)

ALS caused by mutant SOD1 lacks effective and durable therapy. Here, the authors show that a single intravenous AAV9-delivered amiR-SOD1 robustly silences hSOD1 in the CNS and peripheral tissues, preserves neuromuscular function, and markedly extends survival in ALS mice.

## Introduction

Amyotrophic lateral sclerosis (ALS) is a fatal neurodegenerative disease, typically leading to progressive paralysis, respiratory failure, and death within 2–5 years of symptom onset^1,2^. While most ALS cases are sporadic, approximately 10% are familial. Autosomal dominant mutations in the human superoxide dismutase 1 (h*SOD1*) gene account for 10–20% of familial and ~2% of sporadic ALS cases^3–5^. Mutant hSOD1 proteins are known to be conformationally unstable and prone to aggregation. These aggregates contribute to cellular toxicity through multiple pathways, including oxidative stress, mitochondrial dysfunction, endoplasmic reticulum stress, impaired axonal transport, and autophagy disruption^6–8^. Although ALS is primarily characterized by motor neuron loss, mutant hSOD1 expression in non-neuronal cells, such as astrocytes and microglia, also accelerates disease progression^9,10^. Moreover, peripheral tissues, including skeletal muscle, have been implicated in ALS pathogenesis independently of motor neurons^11–15^. These findings suggest that systemic suppression of SOD1 in both CNS and peripheral tissues may offer enhanced therapeutic benefit.

Therapeutic strategies aimed at suppressing SOD1 expression have been developed to treat mutant SOD1-induced ALS in animals and human patients^16–22^, including antisense oligonucleotides (ASOs), small interfering RNAs (siRNAs), and recombinant adeno-associated virus (rAAV)-delivered short hairpin RNAs (shRNAs) or artificial microRNAs (amiRs)^17–19,23,24^. rAAV vectors transfer genes into cells in the CNS and peripheral tissues, providing long-term transgene expression with minimal pathogenic consequences^25–27^. Preclinical trials have been conducted using rAAV to deliver transgenes expressing shRNAs or amiRs to silence disease-causing, gain-of-toxicity type of gene mutations^28^. However, we discovered that shRNA-encoding sequences generate truncated AAV genomes via template switching during viral vector production, raising significant concerns about the efficiency and consistency of gene delivery using this strategy in humans^29^. Additionally, direct repeats within the vector genome can also result in truncation events through homology recombination during vector production^30^. To solve these problems, we designed a self-complementary AAV-amiR vector, which carries dual amiRs targeting *SOD1* (amiR-*SOD1*). The amiRs are based on miR-33 scaffold due to its favorable stem-loop structure that preserves rAAV genome integrity, reduces off-target effects, and maintains effective gene silencing in vivo^31–36^. The dual amiRs are designed to maximize gene silencing efficacy from a single-vector genome while minimizing AAV genome truncation caused by inverted and direct repeats during vector manufacturing.

The SOD1^G93A^ mouse model recapitulates key clinical and pathological features of ALS, including progressive muscle weakness, respiratory impairment, and motor neuron degeneration^37,38^. It has been well established that a gain of toxicity by the mutant hSOD1 causes motor neuron degeneration and ALS symptoms. To suppress the mutant *SOD1* expression, we employed the human survival motor neuron 1 (hSMN) promoter^39,40^ to drive the dual amiR-*SOD1* expression, because the hSMN1 promoter enables highly efficient neuronal transgene expression in the CNS and has been proven efficacious and safe in AAV-mediated gene replacement therapy for Spinal Muscular Atrophy (SMA)^41^. We packaged the vector into AAV9^42,43^ since it is clinically approved and can transduce both the CNS and peripheral tissues, including muscles. Here, we show that a single IV injection of the AAV9-amiR-*SOD1* vector in 60-day-old SOD1^G93A^ mice reduced *SOD1* expression in both CNS and peripheral tissues, preserved respiratory and motor functions, and extended survival by approximately 100 days, representing the most substantial therapeutic benefit from a single IV administration reported to date in this model. Remarkably, therapeutic benefits were also observed when treatment was initiated after symptom onset, strongly supporting the clinical potential of this gene-silencing strategy for ALS.

## Results

### Designing and testing the AAV9-amiR-SOD1 vector

**Fig. 1:**
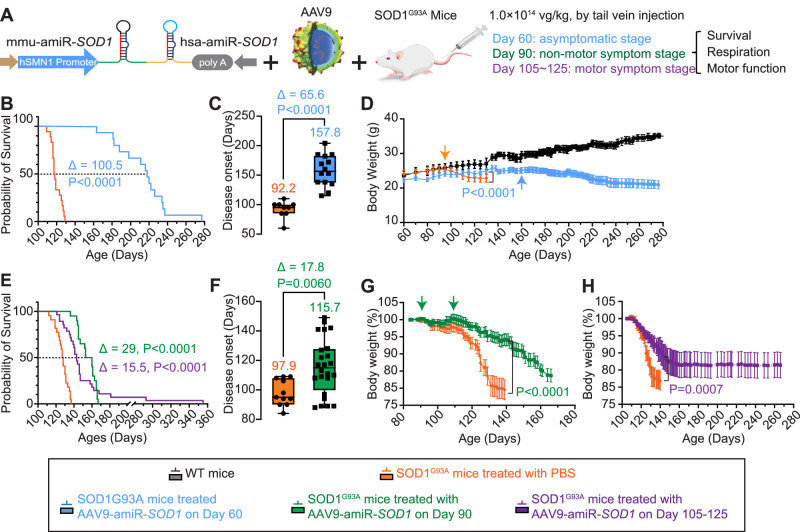
Systemically delivered AAV9-amiR-*SOD1* before and after disease onset improves survival and slows disease progression in SOD1^G93A^ mice. **A** A schematic illustration of AAV9-amiR-*SOD1* genome design and experimental scheme of animal studies. Mmu, mus musculus; hsa, homo sapiens. **B** Kaplan-Meier survival plots of SOD1^G93A^ mice receiving AAV9-amiR-*SOD1* (blue, *n* = 15) or PBS (orange, *n* = 18) on day 60. **C** Disease onset in SOD1^G93A^ mice treated with AAV9-amiR-*SOD1* (blue, *n* = 14) or PBS (orange, *n* = 9) on day 60. Data are shown as mean ± SEM. **D** Body weight changes of SOD1^G93A^ mice treated with AAV9-amiR-*SOD1* (blue, *n* = 14) or PBS (orange, *n* = 10) on day 60. Age-matched WT mice (black, *n* = 7) were used for comparison. The mice were weighed weekly and the average peaks were indicated by arrows. **E** Kaplan-Meier survival plots of SOD1^G93A^ mice receiving AAV9-amiR-*SOD1* on day 90 (green, *n* = 24) or day 105–125 (purple, *n* = 29). **F** Disease onset in SOD1^G93A^ mice treated with AAV9-amiR-*SOD1* (green, *n* = 24) or PBS (orange, *n* = 11) on day 90. **G**, **H** Body weight changes of SOD1^G93A^ mice treated with AAV9-amiR-*SOD1* on day 90 (green, *n* = 24) or day 105–125 (purple, *n* = 29). Data (**D**, **G**, and **H**) are shown as mean ± SEM. The arrow indicates the first and second peaks of body weight. For each boxplot in (**C**, **F**), each individual point corresponds to a single mouse; the central line corresponds to the median (50th percentile); the lower and upper bounds correspond to the first and third quartiles (25th and 75th percentiles), respectively; and the upper and lower whisker lines extend to the largest and smallest values within 1.5x interquartile range, respectively. The statistical significance was determined as follows: Kaplan–Meier survival analysis using the two-sided log-rank test (**B**, **E**); Two-tailed unpaired Student’s t-test with Welch’s correction (**C**, **F**); Two-way ANOVA followed by Bonferroni’s post hoc test (**D**, **G**, and **H**). *P* values are shown between the indicated groups/time points. Source data are provided as a Source Data file.

We designed a self-complementary AAV9 vector expressing dual amiR-*SOD1* silencers under the control of the human *SMN1* promoter (Fig. 1A and Supplementary Fig. 1). The dual amiR-*SOD1* aimed to enhance silencing efficiency in a single vector. However, direct repeats of identical amiRs can lead to truncation events through homology recombination during vector production^30^. To mitigate this problem, we incorporated two distinct amiR-*SOD1* sequences. Both amiRs share the same guide strand that targets the human *SOD1* gene but are embedded in the mouse and human miR-33 scaffolds, respectively. In addition, these two scaffolds have identical stem-loop structures but differ in their flanking sequences (Supplementary Fig. 1). We tested the dual amiR design against single amiR versions and found that it was superior at silencing SOD1, particularly at low vector concentrations (Supplementary Fig. 2).

We also carefully considered promoter selection because it is critical to the safety and efficacy of AAV-mediated gene silencing^44–46^. We previously demonstrated that a 2nd-generation AAV9 gene therapy expressing an h*SMN1* transgene, driven by a promoter derived from the native h*SMN1* gene, outperforms a benchmark vector identical in design to Zolgensma, the FDA-approved AAV9 gene therapy for treating SMA^47^. The hSMN1 promoter enabled highly efficient neuronal transgene expression in the CNS and significantly improved the therapeutic outcomes as demonstrated by lower effective dose, broader therapeutic window, longer lifespan, minimal peripheral tissue pathologies, and preservation of cardiac, respiratory, and motor functions. Additionally, the use of the promoter enhanced safety as shown by no increase in transaminase levels, a marker for liver damage^41^. Those reports led us to utilize the hSMN promoter to drive the amiR expression in the current vector design (Fig. 1A).

### Intravenous administration of AAV9-amiR-SOD1 before and after disease onset slows ALS progression and extends survival in SOD1^G93A^ mice

The dual amiR construct was packaged into AAV9 and injected into the SOD1^G93A^ mice by tail-vein at a dose of 1.0 × 10^14^ vg/kg to a cohort of SOD1^G93A^ mice at day 60 (asymptomatic stage). The dose at 1.0 × 10^14^ vg/kg was chosen because AAV9 at 1.1 × 10^14^ vg/kg by intravenous injection (IV) was used in human patients^47–49^. Phosphate-buffered saline (PBS)-injected SOD1^G93A^ mice served as controls for comparison of therapeutic outcomes. When the treatment was administered on day 60, the median survival of treated mutant mice (*n* = 15; 9 males and 6 females) was extended by 100.5 days compared to the PBS-injected mice (*n* = 18; 11 males and 7 females) (Fig. 1B).

Disease onset in the SOD1^G93A^ mouse model is commonly defined as the age when the body weight peaks^50^. The average body weight peaked on day 92 in PBS-treated SOD1^G93A^ mice; by contrast, the peak was on day 158 in vector-treated mice (Fig. 1C), indicating a 66-day delay in disease onset. Disease progression after onset was also slowed by the vector treatment. From the peak to humane endpoints (~day 130), the PBS group lost body weight at the rate of 0.46 g/week; by contrast, the rate of body weight decline from the peak to the endpoint (~day 220) in vector-treated mice was 0.28 g/week (Fig. 1D), indicating that the vector treatment slowed down the rate of disease progression by 39%. Interestingly, when the vector-treated group reached their endpoint, atypical symptoms such as urinary and gastrointestinal complications contributed to humane euthanasia in ~50% of the mice despite the preserved motor neurons and motor function (see below), whereas all the PBS-treated mice reached their endpoint by typical ALS symptoms such as paralysis or drastic weight loss (Supplementary Fig. 3). To further gauge the therapeutic potency of the AAV9-amiR-*SOD1* vector, we treated the SOD1^G93A^ mice using 3.3 × 10¹³ vg/kg, a dose that was one third of the clinically relevant dose described above. Again, we observed widespread *SOD1* silencing and robust efficacy, delaying the median onset by 30 days and extending survival by 43.5 days (Supplementary Fig. 4A, B).

A major challenge in ALS therapy is achieving efficacy when the treatment is initiated after the disease is diagnosed, because therapeutic options are chosen at this stage in clinics. To evaluate whether the AAV9-amiR-*SOD1* can meet this challenge, we initiated treatment at later disease stages. Separate cohorts of SOD1^G93A^ mice received a single IV dose of AAV9-amiR-*SOD1* (1.0 × 10¹⁴ vg/kg via tail-vein injection) at either day 90 (near onset, *n* = 25; 15 males and 10 females), or between days 105–125 (mid-disease progression, *n* = 29; 16 males and 13 females). These late-stage interventions extended median survival by 29 and 15.5 days, respectively (Fig. 1E). Strikingly, the treatment at day 90 delayed the disease onset by 18 days (Fig. 1F), as shown by a transient reversal of the body weight decline following the treatment, generating a second and higher body weight peak at day 109. From the body weight peak to the endpoint, the rate of weight loss was 0.46 g/week in the PBS-treated group, whereas the rate was 0.37 g/week in the vector-treated group until the median endpoint day 146, representing a 20% slowdown in the rate of disease progression (Fig. 1G). Similarly, in the 105-125-day treatment cohort, from treatment start to the endpoint, the rate of weight loss was 1.2 g/week in the PBS group, whereas it was 0.84 g/week in the vector-treated group, representing a 30% slowdown in disease progression (Fig. 1H). Both female and male SOD1^G93A^ mice responded to treatment before or after disease onset, though females showed better survival (Supplementary Fig. 5**)**.

### AAV9-amiR-SOD1 enables widespread and sustained SOD1 silencing in the CNS and peripheral tissues

**Fig. 2:**
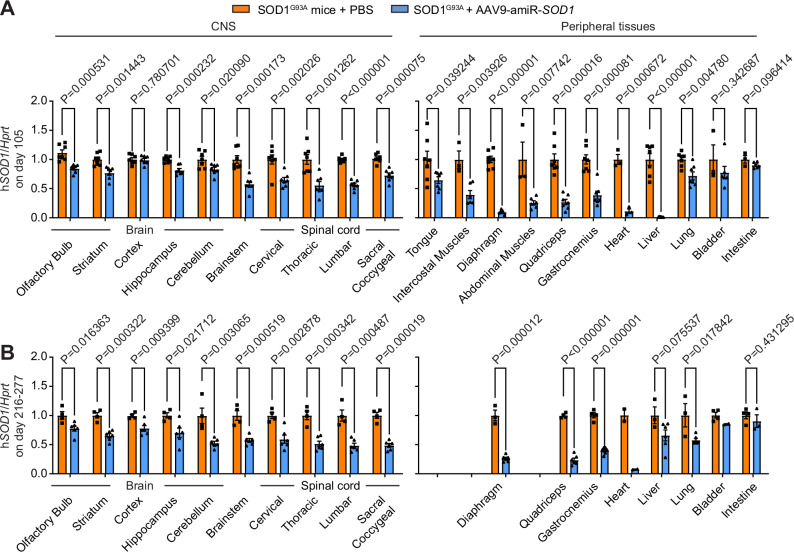
AAV9-amiR-*SOD1* treatment knocks down *SOD1* mRNA expression in the CNS and peripheral tissues. (**A**) h*SOD1* mRNA levels in the CNS and peripheral tissues at day 105 in SOD1^G93A^ mice treated with AAV9-amiR-*SOD1* or PBS on day 60 (*n* = 6–7/group). (**B**) h*SOD1* mRNA levels in the CNS and peripheral tissues at the humane endpoint in SOD1^G93A^ mice treated with PBS or AAV9-amiR-*SOD1* on day 60. The PBS and AAV9-amiR-*SOD1* groups were euthanized on days 118–132 and 216–277, respectively. For analysis in the AAV9-amiR-*SOD1* group, *n* = 6 for the CNS tissues; *n* = 6 for the diaphragm, quadriceps, gastrocnemius, liver, and lung; *n* = 2 for the bladder and heart. In the PBS group, *n* = 4 for the CNS tissues; *n* = 4 for the quadriceps, gastrocnemius, bladder, and intestine; *n* = 3 for the diaphragm, liver, and lung; *n* = 2 for the heart. Data are shown as mean ± SEM. Each individual point corresponds to a single mouse. Multiple unpaired Student’s *t*-test with Welch’s correction was used, and the *P*-value without a line indicates a comparison with the corresponding PBS group. Source data are provided as a Source Data file.

To investigate the molecular basis of the observed therapeutic benefits, we measured human SOD1 expression in both CNS and peripheral tissues using qRT-PCR. Mice were treated on day 60 and then sacrificed on day 105 for tissue collection. Compared to PBS-treated animals, the vector-treated group showed a reduction in the h*SOD1* mRNA levels in CNS and peripheral regions that are crucial for local motion and respiratory functions, including the brainstem, cervical, thoracic, lumbar, and sacral spinal cord, diaphragm, intercostal muscles, abdominal muscles, tongue, and quadriceps and gastrocnemius muscles (Fig. 2A, Supplementary Table 1). Immunohistology staining with a human SOD1 antibody confirmed the downregulation of hSOD1 expression in muscle fibers after AAV treatment (Supplementary Fig. 6). After lowering the dose to 3.3 × 10¹³ vg/kg, h*SOD1* mRNA levels were still reduced in the CNS and peripheral tissues after gene therapy compared to the PBS group. Compared to the 1.0 × 10^14^ vg/kg dose, one-third of the dose achieved similar silencing effects in the CNS, but was less effective in peripheral tissues, particularly in muscles (Supplementary Fig. 4C).

To evaluate long-term silencing, we analyzed tissues collected from mice at their humane endpoints (days 216–277). Sustained h*SOD1* suppression was observed across both the CNS and peripheral tissues, as evidenced by a decrease in h*SOD1* mRNA in multiple regions (Fig. 2B, Supplementary Table 1). Notably, robust h*SOD1* silencing was maintained in the crucial tissues for respiratory and motor functions. In contrast, the liver, which showed ~99% suppression on day 105, exhibited no detectable reduction in h*SOD1* expression at the endpoint, suggesting that AAV genomes were lost over time due to hepatocyte turnover. We also did not detect downregulation of h*SOD1* in the bladder or intestine on either day 105 or the endpoint (Fig. 2B, Supplementary Table 1). AAV9-amiR-*SOD1* did not alter the expression levels of mouse endogenous Sod1 as no change was detected in the levels of *Sod1* mRNA in the lumbar spinal cord or diaphragm (Supplementary Fig. 7), consistent with our in vitro experimental data showing that the amiR-*SOD1* selectively targeted the human *SOD1* gene (Supplementary Fig. 8). In summary, systemic delivery of AAV9-amiR-*SOD1* efficiently and persistently silenced human *SOD1* in both CNS and peripheral tissues.

### AAV9-amiR-SOD1 treatment preserves the respiratory function

**Fig. 3:**
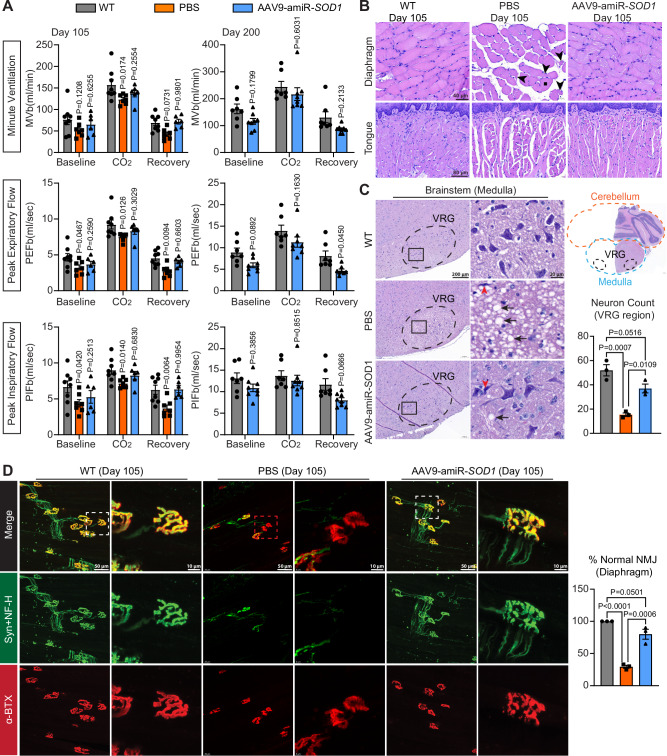
AAV9-amiR-*SOD1* treatment improves the respiration function in the SOD1^G93A^ mice. **A** Animal breath was quantified using whole-body plethysmography on day 105 (left) (Age-matched WT mice, *n* = 8; PBS-treated SOD1^G93A^ mice, *n* = 7; AAV9-amiR-*SOD1*-treated SOD1^G93A^ mice, *n* = 6) and day 200 (right) (Age-matched WT mice, *n* = 7; AAV9-amiR-*SOD1*-treated SOD1^G93A^ mice, *n* = 8). The SOD1^G93A^ mice were treated with AAV9-amiR-*SOD1* vector or PBS on day 60. Age-matched WT mice were used for comparison. The PBS-treated SOD1^G93A^ mice did not survive on day 200 for the test. Data are shown as mean ± SEM. *P*-values are from the comparison with the corresponding WT group. One-way ANOVA followed by Bonferroni’s post hoc test (Day 105); Two-tailed unpaired Student’s *t*-test with Welch’s correction (Day 200). **B** Representative H&E staining of diaphragm (top) and tongue (bottom) tissue sections (*n* = 3 mice per group, with 3 images analyzed per mouse). Mice were treated on day 60 and tissues were collected on day 105. Arrowheads, atrophic fibers. **C** H&E staining of brainstem sections. The number of neurons in the VRG region was quantified (*n* = 3 mice per group). Dotted circles mark VRG regions; black arrows point to vacuoles; and red arrowheads indicate atrophic neurons. Data are shown as mean ± SEM. Comparison was analyzed by one-way ANOVA. **D** The diaphragm muscle sections from mice treated on day 60 and collected on day 105 were immunostained with α-BTX/SYN/NF-H to visualize NMJs. The dashed-line boxes mark the areas that were enlarged and shown to the right, providing a clear view of the absence of axonal terminal occupation in the post-synaptic NMJs in PBS-treated mice and the complete occupation in wild-type and vector-treated mice. The white and red boxes represent intact and damaged NMJ, respectively. The bar graph shows the quantified percentages of NMJs occupied by axonal terminals (mean ± SEM, *n* = 3 mice per group). Comparison was analyzed by one-way ANOVA. Source data are provided as a Source Data file.

Respiratory failure is the most frequent cause of ALS patient death^51–53^, and respiratory support significantly prolongs survival^51,54^. SOD1^G93A^ mice exhibit significant respiratory insufficiency, restrictive lung disease, and hypoventilation^55^. A previous report showed that local administration of an AAV vector expressing amiR against hSOD1 in the tongue and intrapleural space of SOD1^G93A^ mice improved breathing function and prolonged lifespan^56^. We therefore treated mice on day 60 and assessed breathing function on days 105 and 200 using whole-body plethysmography to evaluate minute ventilation (MV), peak expiratory flow (PEF), and peak inspiratory flow (PIF) in each of three phases: baseline, CO_2_ challenge, and recovery. Compared to age-matched WT littermates on day 105, PBS-treated SOD1^G93A^ mice exhibited similar MV at baseline but significantly lower MV when challenged with CO_2_ and during recovery. Additionally, PBS-treated mice exhibited impaired PEF and PIF at baseline, and during CO_2_ challenge and recovery compared with their WT littermates (Fig. 3A). In contrast, AAV9-amiR-*SOD1*-treated SOD1^G93A^ mice performed similarly in MV, PEF, and PIF to the age-matched WT littermates in all three phases (Fig. 3A, left panels). By day 200, all PBS-treated mice died of the disease, but the vector-treated mice survived, although their respiratory function was measurably lower in five of the nine parameters (Fig. 3A, right panels). The improvement in respiratory function produced by this AAV9-amiR-*SOD1* vector is more pronounced than that of previously reported vectors^44,56,57^, and likely contributed to the extended survival of these mice.

To investigate the anatomical basis of these physiological improvements, we performed histological analysis. Hematoxylin and eosin (H&E) staining of the diaphragm tissue sections from the 105-day-old mice revealed small angular atrophic myofibers in the PBS-treated group but not in the AAV-amiR-*SOD1*-treated ones (Fig. 3B, top; Supplementary Fig. 9). The PBS-treated SOD1^G93A^ mice also exhibited severe atrophy in the tongue muscles compared to WT littermates, but the vector-treated group did not (Fig. 3B, bottom). In the ventral respiratory group (VRG) in the medulla, a key area for breathing control, α-motor neurons were largely lost in the PBS group, and the few that remained were frequently atrophic (Fig. 3C, red arrowheads). In contrast, AAV9-amiR-*SOD1* treatment prevented the motor neuron loss, although some were atrophic (Fig. 3C). Additionally, vacuoles that originated from degenerative mitochondria^58,59^ were conspicuous in the VRG region of the PBS-treated group but nearly absent in the vector-treated group (Fig. 3C, black arrows).

The α-motor neurons in the brainstem and spinal cord transmit signals to muscle fibers via neuromuscular junctions (NMJs), which show denervation in ALS. We assessed NMJs' integrity in the diaphragm by staining the presynaptic NMJs with antibodies to synapsin and neurofilament heavy chain (NF-H), and the postsynaptic NMJs with α-bungarotoxin (α-BTX). WT littermates exhibited 90%-100% colocalization of pre- and post-synapse signals, whereas PBS-treated SOD1^G93A^ mice showed only 0%-20% colocalization, indicating severe denervation. The AAV9-amiR-*SOD1* treatment restored NMJs from denervation, as evidenced by a pattern similar to that of the WT littermates (Fig. 3D). Overall, the AAV9-amiR-*SOD1* treatment improved respiratory function in SOD1^G93A^ mice by protecting the muscles critical for breathing, motor neurons in the respiratory control center, and NMJs in essential respiratory muscles from hSOD1-induced toxicity.

### AAV9-amiR-SOD1 treatment improves limb motor function and muscle strength

**Fig. 4:**
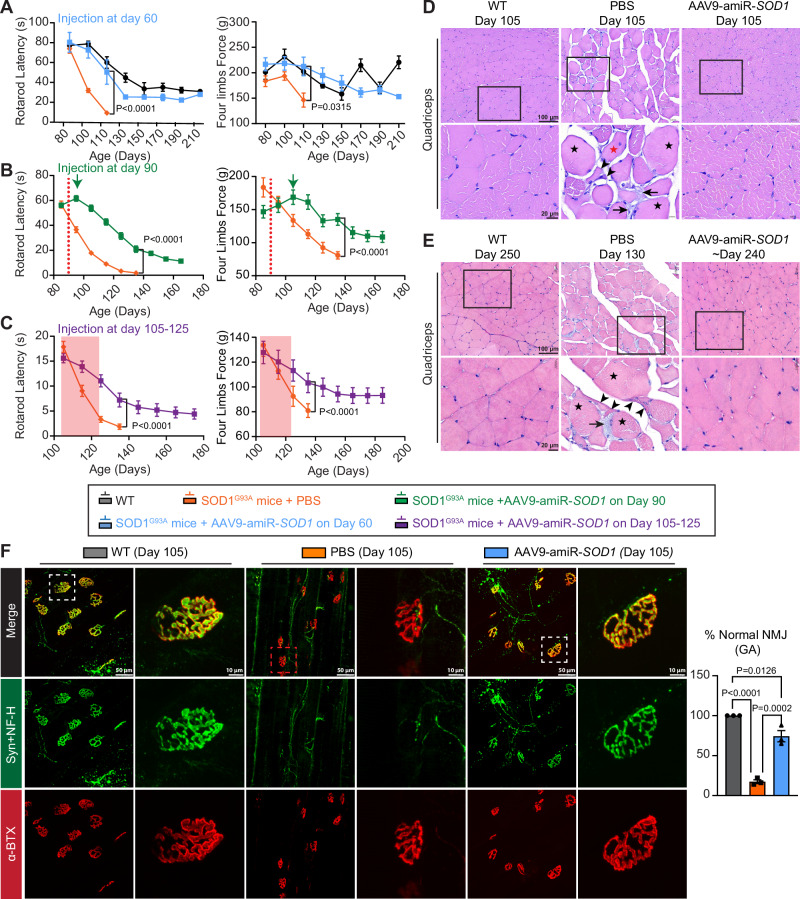
AAV9-amiR-*SOD1* treatment improves motor function and muscle strength in the SOD1^G93A^ mice. **A** Rotarod test and four-limb grip strength tests of SOD1^G93A^ mice treated with AAV9-amiR-*SOD1* (*n* = 11 mice per group) or PBS (*n* = 9 mice per group) on day 60. WT mice (*n* = 9 mice per group) were used as controls. **B** The same tests of SOD1^G93A^ mice as in (**A**), treated with AAV9-amiR-*SOD1* (*n* = 25 mice per group) or PBS (*n* = 8 mice per group) on day 90. The vertical dotted lines indicate the injection time. The arrows indicate the rebound of performances of the rotarod test and grip strength following treatment in the vector-treated SOD1^G93A^ mice. **C** The same tests of SOD1^G93A^ mice as in **A**, treated with AAV9-amiR-*SOD1* (*n* = 29 mice per group) or PBS (*n* = 8 mice per group) on day 105–125. The red-shaded area indicates the age window during which each mouse was treated. Data are shown as mean ± SEM in (**A-C**). **D** Representative H&E-stained quadriceps muscle sections that were treated on day 60 and collected on day 105 (*n* = 3 mice per group). **E** Same as in (**D**) but collected at the endpoint (*n* = 3 mice per group). Stars indicate hypertrophic fibers; arrowheads point to atrophic fibers; the red star indicates a fiber with a centralized nucleus; and arrows point to endomysial fibrosis. **F** The gastrocnemius muscle sections from mice treated on day 60 and collected on day 105 were immunostained with α-BTX/SYN/NF-H to visualize NMJs. The dashed-line boxes mark the areas that were magnified and shown to the right, providing a clear view of the absence of axonal terminal occupation at post-synaptic NMJs in PBS-treated mice and the complete occupation in wild-type and vector-treated mice. The white and red boxes represent intact and damaged NMJ, respectively. The bar graph shows the quantified percentages of NMJs occupied by axonal terminals (mean ± SEM, *n* = 3 mice per group). The statistical significance was determined as follows: Two-way ANOVA followed by Bonferroni’s post hoc test (**A**–**C**); One-way ANOVA followed by Bonferroni’s post hoc test (**F**). *P* values are shown between the indicated groups/time points. Source data are provided as a Source Data file.

In addition to enhancing respiratory function, we evaluated whether AAV9-amiR-*SOD1* treatment also improved limb motor function and muscle strength. The SOD1^G93A^ mice were injected with AAV9-amiR-*SOD1* or PBS on day 60, day 90, or days 105–125, as shown in Fig. 1A. Motor function was analyzed using the rotarod test, and muscle strength was measured by grip force every 10–20 days until reaching the humane endpoint (Fig. 4A–C). The AAV9-amiR-*SOD1* treatment preserved motor function and muscle strength at each intervention time point compared with the PBS group (Fig. 4A–C). Similar to the reversal of body weight decline, administering AAV near disease onset on day 90 transiently improved motor function and muscle strength (Fig. 4B, arrows). The treatment initiated after disease onset, on day 105-120, also slowed the decline in motor function and muscle strength (Fig. 4C).

To determine whether the muscle protection contributed to the improvement on the motor functions, we examined the quadriceps of SOD1^G93A^ mice treated at day 60 and harvested at 105–120 (Fig. 4D) or 237–250 days old (Fig. 4E). In the PBS-treated SOD1^G93A^ mice, the muscle fibers showed hallmarks of denervation, including hypertrophic (black stars) and atrophic fibers (arrowheads), centralized nuclei (red stars), and endomysial fibrosis (arrows). In contrast, the AAV9-amiR-*SOD1* treatment preserved normal muscle morphology throughout the study until the humane endpoint (Fig. 4D, E). Next, we examined the NMJs in the gastrocnemius muscle. Similar to the NMJs in the diaphragm, the PBS-treated SOD1^G93A^ mice developed denervated NMJs, while AAV9-amiR-*SOD1* treatment maintained NMJ formation close to the level of their WT littermates (Fig. 4F). Together, these data indicate that AAV9-amiR-*SOD1* treatment preserved limb motor function and muscle strength by maintaining NMJ integrity when administered either before or after disease onset.

### AAV9-amiR-SOD1 treatment protects motor neurons and mitigates spinal cord Inflammation

**Fig. 5:**
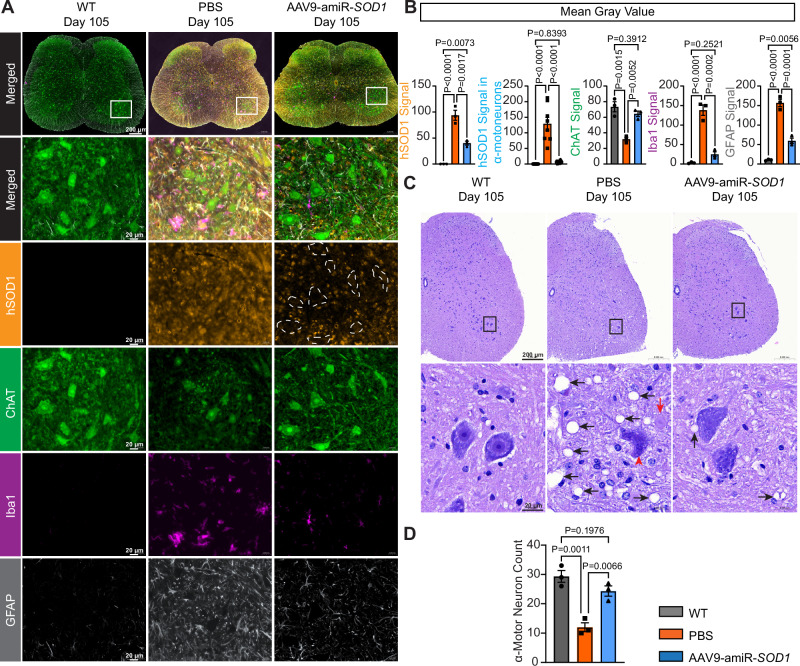
AAV9-amiR-*SOD1* treatment preserves motor neurons and mitigates neuroinflammation in the spinal cord in early disease stages. **A** Lumbar spinal cord sections from mice treated on day 60 and collected on day 105 were analyzed by immunofluorescence using markers as indicated. Age-matched WT mice were used for comparison. The boxed areas in the top row were enlarged in the rows below. The white dashed lines mark the α-motor neurons that were revealed by ChAT staining. **B** Quantification of staining intensity of hSOD1, ChAT, Iba1, and GFAP in sections from each group (*n* = 3 mice per group). The staining intensity of hSOD1 in α-motor neurons was independently quantified for each neuron (*n* = 8–11 neurons in each group, each individual point corresponds to a single neuron). Data are shown as the mean ± SEM. **C** Representative H&E-stained lumbar spinal cord sections (three mice in each group). The black boxes in the top row indicate the areas magnified in the rows below. Black arrows point to vacuoles, the red arrow points to a glassy axonal spheroid, and the red arrowhead points to atrophic neurons. **D** Quantification of the α-motor neurons in lumbar spinal cord sections is shown as the number of motor neurons per section (mean ± SEM, *n* = 3 mice per group). One-way ANOVA followed by Bonferroni’s post hoc test was used, and *P* values are shown between the indicated groups. Source data are provided as a Source Data file.

To assess the cellular distribution of hSOD1 protein in the lumbar spinal cord after gene therapy, we performed immunostaining analysis on spinal cord sections from mice injected at day 60 and harvested on day 105. The overall human SOD1 signal decreased in the vector-injected mice compared with the PBS-treated ones, with hSOD1 expression nearly absent in the choline acetyltransferase (ChAT) positive motor neurons (Fig. 5A, B). Compared to age-matched WT littermates, we observed a loss of ChAT(+) neurons in PBS-treated mice, but this loss was prevented in the vector-treated group. The PBS-treated mice showed an increase in cells positive for Ionized calcium-binding adapter molecule 1 (Iba1) and Glial Fibrillary Acidic Protein (GFAP), indicating the occurrence of neuroinflammation. In contrast, the vector treatment attenuated the increase in Iba1 and GFAP expression, indicating decreased neuroinflammation (Fig. 5A, B). However, the vector treatment had minimal impact on hSOD1 levels in GFAP-positive cells (Supplementary Fig. 10), suggesting that the reduction in neuroinflammation is not directly attributable to hSOD1 knockdown in glia.

The vector treatment also improved other neuropathologies in the spinal cord. At age 105 days, H&E staining revealed frequent axonal spheroids with large glassy cytoplasm (Fig. 5C, red arrow) that are focal swellings along degenerating axons^60^, atrophic neurons (Fig. 5C, red arrowhead) and abundant vacuoles (Fig. 5C, black arrows) in the anterior horn of the PBS-treated SOD1^G93A^ mice. These pathologies were largely eliminated in the vector-treated mice (Fig. 5C). Consistent with the immunofluorescence data that AAV9-amiR-*SOD1* preserved ChAT(+) neurons (Fig. 5A, B), quantification of the large α-motor neurons from the H&E-stained sections confirmed that the vector treatment prevented motor neuron loss (Fig. 5C, D).

**Fig. 6:**
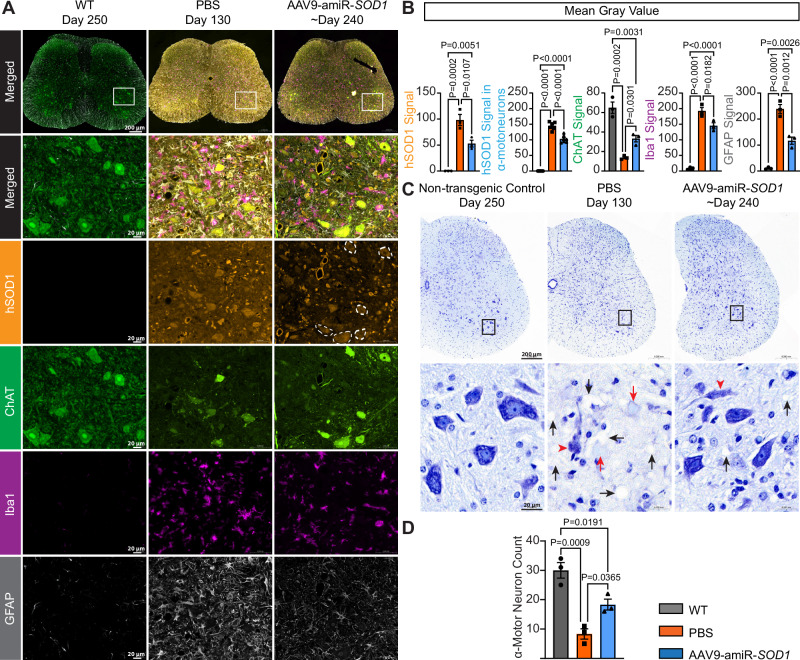
AAV9-amiR-*SOD1* treatment preserves motor neurons and mitigates neuroinflammation in the spinal cord at the disease endpoint. **A** Lumbar spinal cord sections from mice treated on day 60 and collected at the endpoint (day ~130 for the PBS-treated group and ~240 for the vector-treated group) were analyzed by immunofluorescence using markers as indicated. Age-matched WT mice were used for comparison. The boxed areas in the top row were enlarged in the rows below. The white dotted lines mark the α-motor neurons revealed by ChAT staining. **B** Quantification of the staining intensity of hSOD1, ChAT, Iba1, and GFAP in lumbar spinal cord sections of each group (*n* = 3 mice per group). The staining intensity of hSOD1 in α-motor neurons was independently quantified for each neuron (*n* = 6–9 neurons in each group, each individual point corresponds to a single neuron). Data are shown as mean ± SEM. **C** Representative Nissl-stained lumbar spinal cord sections. Black arrows point to vacuoles, the red arrow points to a glassy axonal spheroid, and the red arrowhead points to atrophic neurons. **D** Quantification of the α-motor neurons in lumbar spinal cord sections is shown as the number of motor neurons per section (mean ± SEM, *n* = 3 mice per group). One-way ANOVA followed by Bonferroni’s post hoc test was used, and *P* values are shown between the indicated groups. Source data are provided as a Source Data file.

Remarkably, even at the humane endpoint (~day 240) in the vector-treated group, hSOD1 expression was still suppressed, the ChAT(+) cells persisted, and Iba1 and GFAP signals were kept at lower levels in the lumbar spinal cord, compared with the PBS-treated animals at their humane endpoint (~day 130) (Fig. 6A, B). Furthermore, Nissl staining revealed a significant loss of α-motor neurons in the PBS-treated mice. The few that remained were atrophic (Fig. 6C, red arrowheads), accompanied by frequent axonal spheroids (Fig. 6C, red arrows) and abundant vacuoles (Fig. 6C, black arrows). By comparison, the vector-treated mutant mice at their endpoint (~day 240) still exhibited more surviving motor neurons with normal morphology and fewer vacuoles than the PBS-treated mice at 130 days (Fig. 6C, D). In summary, AAV9-amiR-*SOD1* treatment downregulated hSOD1 expression in motor neurons, alleviated neuroinflammation, and preserved motor neurons in the spinal cord.

### amiR-SOD1 is accurately processed and does not disturb the endogenous miRNA population

**Fig. 7:**
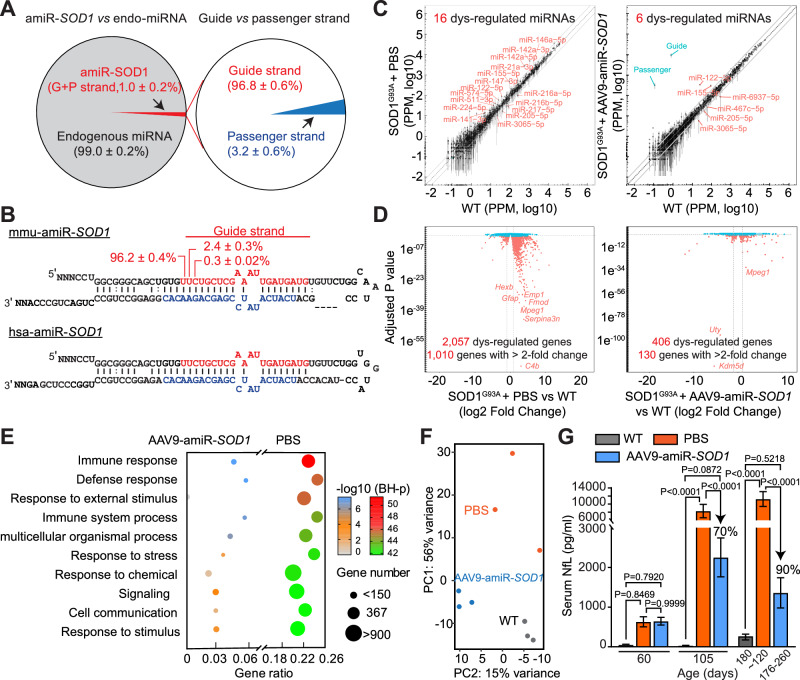
AAV9-expressed amiR-*SOD1* transcripts are processed accurately and normalize the transcriptome and Nf-L levels in the SOD1^G93A^ ALS model. SOD1^G93A^ mice were treated with PBS or AAV9-amiR-*SOD1* on day 60 and analyzed at day 105. Age-matched WT mice were used for comparison. Lumbar spinal cord RNA was extracted and used to profile the transcribed amiR-*SOD1*, miRNAs, and global gene expression. **A** The abundance of the guide and passenger strands of amiR-*SOD*1. **B** Profiling of 5′ of guide (red) strands of amiR-*SOD1* detected in mice by small RNA-seq (mean ± SD, *n* = 3). The guide strands are indistinguishable between the mmu- and hsa-amiR-*SOD1*, and it is assumed that both miR-33 scaffolds contributed to the detected guide strand. **C** Scatterplots comparing the abundance of endogenous miRNAs and small RNAs between WT mice (x-axis) and SOD1^G93A^ mice treated with PBS (y-axis, left plot) or AAV9-amiR-*SOD1* (y-axis, right plot). Each point represents the normalized miRNA reads in parts per million (ppm), averaged from three biological replicates. Error bar represents SD (*n* = 3). Differentially expressed miRNAs (adjusted *P* < 0.05) are in red. The guide and passenger strands are indicated. **D** Comparison of mRNA abundance in whole spinal cord transcriptome analysis. Significantly dysregulated mRNAs are represented in red. *n* = 3 in each group. **E** Gene ontology (GO) analysis showing the top biological processes changes resulting from AAV9-amiR-*SOD1* (left) or PBS (right) treatment in SOD1^G93A^ mice, compared to WT mice. **F** Principal components analysis of the spinal cord transcriptome. **G** Serum Nf-L levels in SOD1^G93A^ mice treated with PBS or AAV9-amiR-*SOD1* on day 60 and analyzed at the indicated ages (*n* = 5–8 mice in each group, each individual point corresponds to a single mouse). Data are shown as mean ± SEM. Two-way ANOVA followed by Bonferroni’s post hoc test was used in (**G**), and (**P**) values are shown between the indicated groups. Source data are provided as a Source Data file.

Our data demonstrate that the AAV9-amiR-*SOD1* achieves robust hSOD1 silencing and provides unprecedented therapeutic benefits in the SOD1^G93A^ mice (Figs. 1–4). However, high levels of AAV-delivered shRNA can disrupt the RNA interference machinery of the host cell, leading to dysregulation of endogenous miRNAs and cellular toxicity^61,62^. Additionally, the efficient and precise processing of the expressed miRNA precursor into the mature therapeutic guide strand is a critical determinant of potency and safety. To assess amiR-*SOD1* RNA processing and its potential interference with endogenous miRNA levels, we sequenced small RNAs from the lumbar spinal cord of mice treated with the AAV9-amiR-*SOD1* vector on day 60 and harvested on day 105. We noticed that, first, the amiR-*SOD1* guide strand was processed accurately as reflected in the composition of the guide and the passenger strands (97% vs. 3%) (Fig. 7A). Second, the 5′ end of the amiR-*SOD1* guide strand was processed with a 96% accuracy even though they were from two different miR-33 scaffolds (Fig. 7B). Third, we observed no evidence for interference of endogenous miRNA processing. The AAV9-delivered amiR-*SOD1* accounted for only 1% of the total miRNA (Fig. 7A), a level far below the previously reported levels required to saturate the endogenous miRNA machinery^16,61–63^. Indeed, further analysis of miRNA profiles revealed only six dysregulated miRNAs (relative to WT mice, with a > 2-fold change and FDR < 0.05) in vector-treated mice, which compared favorably with the 16 detected in PBS-treated SOD1^G93A^ mice (Fig. 7C).

### AAV9-amiR-SOD1 treatment normalizes the transcriptome and the ALS biomarker Nf-L in SOD1^G93A^ mice

To further assess the impact of the treatment, we conducted transcriptomic analysis. Compared to WT mice, SOD1^G93A^ mice exhibited 2,057 dysregulated genes (1010 genes > 2-fold change) in the lumbar spinal cord (Fig. 7D). Most dysregulated genes are involved in neuroinflammation and neurodegeneration (e.g., *C4b*, *Serpina3n*, *Mpeg1*, *Fmod*, *Hexb*, and *Gfap*)^64–67^. Remarkably, AAV9-amiR-*SOD1* treatment reversed most of the dysregulation, yielding 406 dysregulated genes with only 110 genes showing >2-fold change (Fig. 7D). Compared to the WT mice, pathway analysis revealed the dysregulated genes are enriched in immune responses, defense responses, and responses to external stimulus, etc. in the SOD1^G93A^ mice treated with PBS (Fig. 7E, right). However, such enrichments were significantly attenuated in the AAV9-amiR-*SOD1*-treated mice (Fig. 7E). Principal component analysis confirmed that AAV9-amiR-*SOD1* treatment shifted gene expression profiles in SOD1^G93A^ mice toward the WT control pattern, underscoring the broad therapeutic effect of hSOD1 silencing (Fig. 7F).

Neurofilament light chain (Nf-L) is elevated in biofluids, such as blood, in people with ALS as a consequence of axonal degeneration, and this is being used as a biomarker for ALS disease progression in clinical trials. Therefore, we monitored serum Nf-L levels in the treated mice. Compared to WT mice, serum Nf-L levels were significantly higher and increased over time from day 60 to ~day 120 in the SOD1^G93A^ mice (Fig. 7G). AAV9-amiR-*SOD1* treatment decreased serum Nf-L levels by ~70% compared with PBS-treated mice (PBS: 8283 ± 4424 pg/mL; AAV-amiR-*SOD1*: 2,253 ± 1297 pg/mL) at day 105. Compared with PBS-treated SOD1^G93A^ mice at their endpoint (~120 days, 11,253 ± 4113 pg/mL), AAV9-amiR-*SOD1* treatment lowered serum Nf-L levels by ~90% when measured at much older ages (176–260 days, 1359 ± 1080 pg/mL). To assess safety, we measured serum alanine aminotransferase (ALT) and aspartate aminotransferase (AST) to monitor liver function in the treated mice and observed no abnormalities (Supplementary Fig. 11). Collectively, our results indicate that the IV administration of the AAV9-amiR-*SOD1* vector is efficacious and safe.

## Discussion

In this study, we developed an AAV9 vector that combines an optimized miR-33 scaffold^35^ with an endogenous hSMN1 promoter^41,68^. A single intravenous administration of this vector achieved a widespread, efficient, accurate, and durable gene silencing of the mutant gene in SOD1^G93A^ ALS mice. Administration during the presymptomatic stage preserved spinal motor neurons and muscle innervation, thereby dramatically slowing the deterioration of respiratory and other motor functions and substantially prolonging the lifespan of this ALS mouse model. Notably, the treatment almost eliminated mutant hSOD1 protein expression in motor neurons after 45 days (Fig. 5A, B) and maintained effective suppression until humane endpoints in ALS mice **(**Fig. 6A, B**)**. At the same time, motor neurons remain well preserved in ALS mice, as in WT mice, at the early disease stage (Fig. 5C, D) and persisted at humane endpoints **(**Fig. 6C, D**)**. The NMJs in the diaphragm and GA muscle were severely damaged in the ALS mice, but the therapy restored their health to levels close to those in WT mice (Figs. 3D and 4F). Remarkably, administration near the disease onset delayed disease onset, as evidenced by a transient reversal of declines in body weight, motor function, and muscle strength (Figs. 1G and 4B). Even administration after disease onset significantly slows disease progression and extends survival. In addition to the therapeutic efficacy, no treatment-related side effects were observed. These therapeutic benefits from a single IV administration are unprecedented among gene-therapy approaches in this mouse model, indicating that further clinical evaluation is warranted.

Several design elements of our vector may have contributed to the efficacy and safety outcomes of this study. First, we incorporated two distinct amiR-*SOD1* sequences into this self-complementary AAV9 vector to maximize silencing potency (Supplementary Fig. 1). This is essential because in vivo AAV transduction remains limited in the CNS. Both amiRs share the same guide strand that targets the human *SOD1* gene, but each is embedded separately within the mouse and human miR-33 scaffolds. The two scaffolds have identical stem-loop structures but differ in their flanking sequences. This design maximizes the yield of the specific guiding strand targeting *SOD1* mRNA following cell transduction, while reducing the risk of vector genome truncations associated with hairpin structures^29,35^ and direct repeats during vector manufacturing^30^. Second, the two miR-33 scaffolds were accurately processed and yielded the target-specific guiding strand predominantly **(**Fig. 7A, B**)**. This efficient processing likely contributed to the high silencing potency against SOD1 with minimal disturbance to the endogenous miRNA profile (Fig. 7C), and potentially lower off-targeting effects on the transcriptome (Fig. 7D). Third, we used the human SMN1 promoter, which may be advantageous compared to the commonly used cytomegalovirus enhancer/chicken beta-actin (CMVen/CB) promoter^41^. The hSMN1 promoter is highly efficient in the CNS, and many ALS-associated tissues, and being an endogenous mammalian promoter may also have contributed to the long-lasting silencing potency (Figs. 2, 5, and 6). Fourth, we employed IV administration, which enabled suppression of hSOD1 across the CNS and skeletal muscles.

Our observed therapeutic efficacy in the ALS mouse model compares favorably to the previously reported preclinical trials that showed modest therapeutic effects and survival extension^21,44,57,69–74^. As of now, the first and only AAV-delivered h*SOD1*-silencing treatment in a clinical trial demonstrated therapeutic potential in ALS patients^17^. In the preclinical experiment in SOD1^G93A^ mice, a one-time IV administration between days 50-68, this vector extended survival by 22 days but did not delay disease onset^44^. By comparison, a single IV administration of our vector on day 60 extended survival by 100.5 days, delayed disease onset by 65.6 days, and preserved respiratory and motor functions in SOD1^G93A^ mice. Importantly, administration of our vector on day 90, only two days prior to the disease onset in the PBS-treated cohort, extended survival by 29 days and delayed the disease onset by 18 days. Even administered at the mid-disease progression stage (day 105-125), our vector treatment extended the animal survival by 15.5 days **(**Fig. 1E**, F)**. Moreover, measured by the onset delay and survival extension, our vector treatment outperforms other therapies in the SOD1^G93A^ model, including AAV-based gene silencing strategies, growth factor therapies, and antibody-based interventions^69^. Thus, our results reinforce the promise of the AAV-delivered gene-silencing approach for SOD1-ALS and possibly other pathological conditions caused by dominant, gain-of-toxicity-type gene mutations.

Compared with ASO gene silencing approaches, our AAV-delivered gene silencing results also demonstrate advantages. For example, Tofersen is currently approved by the US Food and Drug Administration (FDA) for the treatment of SOD1-ALS. It is a key benchmark for new approaches to SOD1 suppression in terms of therapeutic efficacy and safety. In its clinical trial, Tofersen failed to meet its primary endpoint of improving disease progression or survival. Preclinical studies have shown that when administered via two intracerebroventricular (ICV) injections on days 50 and 94, Tofersen extended the survival of SOD1^G93A^ mice by 37 days^75^. Clinically, Tofersen dosing regimen is demanding. The maximally tolerated dose is 100 mg every two weeks for three doses, followed by an additional 100 mg injection every 28 days^18^. Therefore, repeated dosing through intrathecal injection can be challenging for patients, especially those at a late disease stage with severe physical limitations^76,77^. Tofersen therapy is also constrained by safety concerns due to common side effects, including procedural pain, headache, limb pain, falls, and back pain. Approximately 7% of VALOR study participants experienced serious adverse events, including autoimmune reactions like myelitis, meningitis, and intracranial hypertension^18^. These limitations underscore the need for alternative gene silencing approaches. The strong and long-lasting therapeutic benefits and minimal adverse effects of the AAV vector developed in this report, as well as other alternative approaches^20,78^, offer avenues for further development to expand the clinical treatment repertoire.

Serum neurofilament light chain (sNf-L) level is a key biomarker for neuronal damage, as it played an essential role in the FDA approval of Tofersen^18,19^. In patients, Tofersen treatment led to a 62% average reduction in sNf-L (range: 36%–84%) compared with pretreatment levels after five months. In SOD1^G93A^ mice, Tofersen administration by ICV injection reduced the serum neurofilament heavy chain (sNf-H) by ~50%^75^. By contrast, our IV-administered AAV9-amiR-*SOD1* vector consistently reduced sNf-L by 70-90% throughout the study in the same model (Fig. 7G), indicating robust neuroprotection.

A critical consideration for gene silencing approaches is safety. This is especially true for AAV-delivered therapy, as the treatment cannot be paused or withdrawn once delivered. A recent study reported that delivery of AAV-RNAi vectors targeting *SOD1* into cerebrospinal fluid (CSF) led to elevated serum Nf-H levels and dorsal root ganglion toxicity in mice and NHPs, suggesting potential harmful side effects^79^. The safety of our vector will require further detailed study in NHPs. Nevertheless, our data have shown an encouraging safety profile in the current study. The amiR-*SOD1* was accurately processed to generate the specific guide strand and strongly suppressed mutant *SOD1* expression but did not perturb the endogenous miRNA expression pattern in the spinal cord (Fig. 7C). Furthermore, the vector treatment normalized the gene expression profile from the perturbed pattern of the mutant spinal cord to a near wild-type pattern (Fig. 7E), suggesting little if any off-target effect. Additionally, we observed no evidence of liver toxicity based on the levels of AST and ALT (Supplementary Fig. 11). The IV administration route, rather than IT or ICV administration in the prior study, could also be a contributing factor for the safety profile that we observed in our study^79,80^.

Despite the robust efficacy of the AAV9-amiR-*SOD1* vector, further improvement may be achievable, for example, by enhancing *SOD1* silencing in the cerebral cortex and in astrocytes. Additionally, the SOD1^G93A^ mouse model exhibits urinary dysfunction and intestinal motility dysfunction. Urinary system dysfunction is detectable before the onset of motor symptoms and progresses to severe urinary retention and bladder overdistension in late disease stages^81^. SOD1^G93A^ mice also show a diminished intestinal peristalsis, which is accompanied by a significant reduction in smooth muscle myosin heavy chain^82^. The long-term consequences of impaired urinary function and intestinal motility can be fatal. These symptoms are closely associated with the accumulation of misfolded SOD1 protein in the smooth muscle system^82^, suggesting that an enhanced *SOD1* silencing in smooth muscles could further improve outcomes.

Although our experiments have demonstrated robust results with the AAV9-amiR-*SOD1* vector in the SOD1^G93A^ mouse model, it remains unclear whether these results can be translated into human ALS therapy. Previously, many compounds that showed benefits in SOD1^G93A^ mice have failed in human trials. These compounds targeted potential mechanisms of neurodegeneration but did not directly target the mutant hSOD1 protein. Nevertheless, in human trials, these compounds are often used to treat sporadic ALS^83^. By contrast, our approach specifically suppresses the h*SOD1* gene, with the goal of being translated into individuals with SOD1-ALS. A previous case of success with this approach is ASO Tofersen, which entered clinical trials based on its efficacy in SOD1^G93A^ mice^75^. From a translational perspective, our AAV9-amiR-*SOD1* approach offers advantages over the ASO approach. First, long-term benefits are achieved with a single treatment, eliminating the need for repeated dosing. Second, intravenous administration enables systemic therapy throughout the body and overcomes complications associated with multiple invasive injections (intrathecal, intracerebroventricular, intracisternal magna, or spinal subpial)^21,57,72,84,85^. Many studies have demonstrated that the misfolding and aggregation of the mutant hSOD1 protein lead to cytotoxicity across multiple systems in both the CNS and the periphery^11–15,82^. The toxicity of mutant hSOD1 in peripheral tissues, such as skeletal muscle, can lead to ALS-like pathology in the spinal cord and motor neuron degeneration^14,15^. Therefore, peripheral h*SOD1* silencing, in addition to the CNS, delivered by IV administration of the vector used in this report, may offer additional benefits. Third, AAV9 has undergone extensive evaluation in clinical trials for multiple CNS diseases and has been successfully employed to treat SMA by systemic injection^47,86,87^. The 1.0 × 10^14^ vg/kg dose of AAV9 delivered by IV injection is considered safe for clinical applications in pediatric patients^47^. In adult patients, it is not yet clear whether this dose is appropriate, and a reduced dose may be required. We tested a lower AAV9-amiR-*SOD1* dose, one-third of the clinically used dose, and, encouragingly, observed meaningful benefits in survival and disease onset in SOD1^G93A^ mice (Supplementary Fig. 4), further reinforcing the translational potential of the AAV9-amiR-*SOD1* vector. Additional dose reduction for IV administration may be achieved by utilizing AAV capsids with enhanced CNS tropism^88–93^.

In summary, we showed that a single intravenous administration of an engineered AAV9-gene silencing vector elicits specific silencing of human SOD1 and robust therapeutic efficacy in ALS mice when administered either pre-symptomatically or during the symptomatic stage. Importantly, the robust therapeutic efficacy and minimal adverse effects were substantiated by strong evidence from whole-body h*SOD1* silencing, motoneuron protection, accurate amiR-*SOD1* processing, and normalization of transcriptome and biomarkers. The significant therapeutic benefits observed after disease onset highlight the potential of our approach for clinical application in SOD1-ALS and other disease-causing, gain-of-toxicity gene mutations.

## Methods

### Vector design, construction, and production

The amiR-*SOD1* vector genome is composed of an endogenous h*SMN1* promoter^41^, a bgh-polyA sequence, and two amiRs targeting the human *SOD1* gene embedded in mouse and human *miR-33* scaffolds^35^. The guide strand of amiR-*SOD1* targets the 51-171^th^ nucleotides of the human *SOD1* coding sequence. The construct was incorporated into a self-complementary AAV (scAAV) vector and packaged into AAV9. The rAAVs were produced by transient HEK293 cell (ATCC:CRL-1573) transfection and CsCl ultracentrifugation by the UMass Chan Medical School Viral Vector Core, as previously described^94^. Vector preparations were titrated by digital-droplet PCR (ddPCR), and purity was assessed by 4–12% SDS-polyacrylamide gel electrophoresis and Flamingo staining (Invitrogen).

### Animal procedures

The animal experiments were approved by the Institutional Animal Care and Use Committee (Protocol #: PROTO202000037 and IPROTO202400000060)) of UMass Chan Medical School and conducted in a double-blind manner. Male B6SJL-Tg (SOD1*G93A)1Gur/J mice (JAX: 002726) and female non-transgenic mice (JAX: 100012) of the same genetic background were obtained from Jackson Laboratory. Parental mice were mated in the UMass Chan Medical School Animal Facility. Mice were housed in standard cages in a temperature-controlled room (22–24 °C) with a 12 h dark-light cycle and fed with standard chow (LabDiet, #5P7622; 22.5% protein, 5.4% fat, 4% fiber, 50% polysaccharide). Tail samples were collected from the offspring, and DNA was extracted using KAPA lysis buffer (KAPA Express Extract, Roche, 07961626001). Mice transgene copy numbers were monitored by ddPCR using the primer/probe set provided by Jackson Laboratory. Mice with fewer than 20 *copies of SOD1* were excluded from the study. Mice with similar h*SOD1* transgene copy number were assigned to the PBS- and vector-treated groups when treated on days 60, 90, and 105-125. The SOD1^G93A^ mice were injected IV via the tail vein on day 60, day 90, or during the symptomatic stage (between days 105 and 125). The symptomatic stage was defined based on age and symptoms: (1) male mice aged between 105 and 115 days, or female mice aged between 115 and 125 days; (2) one or both flexed hind limbs, noticeable tremor, or significantly depressed groin. Mice were considered to have reached the symptomatic stage if they met both the age criterion and at least one symptom. Mice were weighed weekly from day 60 to 130, then twice a week to monitor disease onset and progression. Disease onset was defined as the age at which peak body weight occurred, retroactively. The end stage was reached when either of the following conditions was met: a rapid loss of body weight (>20% in a week) or loss of righting reflex, which was defined as when placed on its side, the animal could not right itself within 15 seconds or when severe urination scald developed. At the end stage, animals were euthanized, and tissues were collected for postmortem analysis.

Grip strength was measured by the grip strength meter (BIOSEB, BIO-GS4). The mice were allowed to grab the metal grid or triangular pull bar and were then pulled backward in a horizontal plane. The force applied to the grid or the bar just before loss of grip was recorded as the peak tension. To test four-limb strength, both the forelimb and hindlimb were allowed to hold the metal grid, and then three peak tensions (measured in grams) were recorded for five consecutive trials. Training was completed one week prior to the first formal test. Rotarod tests (IITC Rotarod) were conducted every 20 days starting on day 60 in each group. Mice were trained using an accelerating rotarod apparatus in acceleration mode (4–40 rpm) at a fixed speed of 20 rpm for 5 min prior to testing. On the day of the formal test, mice were placed in the acceleration mode for 5 min each time, and then the latency to fall was recorded. The average latency of three consecutive trials was recorded for each group. Training was completed one week prior to the first formal test.

### Plethysmography

Respiratory function was assessed using unrestrained whole-body plethysmography (WBP) with the FinePointe™ system (Data Sciences International, Buxco, USA). During WBP, each mouse is placed in an individual, airtight chamber where it can move freely and breathe spontaneously. Respiratory cycles produce small pressure changes in the chamber due to compression and expansion of gases in the lungs, as well as changes in temperature and humidity of the inspired and expired air. These fluctuations in the chamber pressure signal are used to derive respiratory parameters like tidal volume and breathing frequency^95–97^. Prior to each recording session, the plethysmography chambers were calibrated using a predefined air volume injection to ensure accurate measurement of tidal volume and airflow parameters. Each mouse was then placed in the chamber and allowed to acclimate for 20 minutes under room air conditions to minimize stress-induced respiratory variability. Following acclimation, a baseline respiratory recording was collected for 10 minutes under room air. Mice were then subjected to a hypercapnia challenge (CO_2_ 7%, O_2_ 21%), administered for 10 minutes. After CO_2_ exposure, mice were returned to room air for a 10-minute recovery period. Respiratory parameters—including minute ventilation (MV), peak inspiratory flow (PIF), and peak expiratory flow (PEF)—were measured at each stage and data were analyzed using FinePointe software with predefined breath-detection settings.

### Tissue collection

Mice were perfused with 20 mL ice-cold PBS under anesthesia with isoflurane inhalation (1-3%). The spinal cord, brain, heart, lungs, liver, diaphragm, intercostal muscles, abdominal muscles, quadriceps, gastrocnemius, tongue, intestines, and bladder were then dissected. The spinal cord was then divided into cervical, thoracic, lumbar, and sacrococcygeal segments. Each tissue was divided into two pieces; one was placed in ice-cold, freshly prepared 4% paraformaldehyde dissolved in 0.15 M sodium phosphate buffer (pH 7.4). Samples other than muscles were fixed overnight at 4 °C and then embedded in paraffin. The other tissue pieces were flash-frozen on dry ice and saved for qRT-PCR.

### Immunostaining analysis

Paraffin sections were deparaffinized and hydrated through xylene (15 min, twice) and ethanol (5 min each at 100%, 100%, and 75%), followed by PBS (5 min, three times). Antigen retrieval was performed by heating the sections in a citrate-based unmasking buffer in a pressure cooker for 15 min, followed by cooling at room temperature for 20 min. Sections were blocked with 2.5% goat serum and incubated overnight at 4 °C with the primary antibody Human SOD1 (Thermo Fisher, MA1-105, 1:1000), followed by TSA-CY3 detection on the next day. On Day 2, after blocking with 3% BSA solution, sections were incubated overnight with the primary antibodies ChAT (MilliporeSigma, ab144p, 1:1000), GFAP (Cell Signaling, 3670, 1:200), and IBA-1 (Cell Signaling, 17198, 1:100). Secondary Donkey anti-Goat FITC secondary antibody for ChAT was applied for 1 hour, followed by additional secondary antibodies: CY7-ms for GFAP and CY5-rb for IBA-1. Slides were counterstained with DAPI using Fluoroshield for imaging. The whole images were acquired using 3D histech MIDI II scanner. Identical camera and fluorescent light source settings were used across all sections and microscopy sessions. hSOD1, ChAT, Iba, and GFAP signal intensity and neuron number were quantified by ImageJ.

### Neuromuscular junction immunofluorescence

The freshly dissected diaphragm was fixed in 4% paraformaldehyde in PBS at room temperature for 15 minutes, then rinsed three times with PBS. The diaphragm was permeabilized with 0.5% Triton X-100 in PBS for 30 minutes, incubated in 1 µg/mL α-BTX (α-bungarotoxin, Alexa Fluor 555 conjugate, Invitrogen, no. B35451) staining solution in PBS for 30 minutes, then in a blocking reagent containing 3% BSA and 0.1% Triton X-100 in PBS for 3 hours, followed by incubation of primary antibodies at 4 °C for 24 hours. The following primary antibodies were used: Synapsin (rabbit, Milipore-Sigma, anti-synapsin I, no. S193, dilution 1:50), Neurofilament (chicken, Milipore-Sigma, anti-neurofilament H, no. AB5539, dilution 1:500). At last, the tissues were rinsed 3 times in PBS for 5 minutes and incubated in a PBS solution containing secondary antibodies (Goat anti-Rabbit IgG (H + L) Highly Cross-Adsorbed Secondary Antibody, Invitrogen, A32731, 1: 1000; Goat anti-Chicken IgY (H + L) Cross-Adsorbed Secondary Antibody, Invitrogen, A32931, 1: 1000) at room temperature for 4 hours. The slides were mounted with Fluoromount-G Mounting Medium (0100-01, Southern Biotech) for imaging with a Leica SP8 microscope. NMJs were defined to be intact (90–100% co-localization), partially denervated (20–90% co-localization), or denervated (0–20% co-localization). For the NMJs in the gastrocnemius muscle, the immunostaining procedure is the same as above, except for the tissue processing. Freshly dissected gastrocnemius muscle was fixed in 4% paraformaldehyde solution in PBS at room temperature for 1 hour, rinsed 3 times with PBS, and then small muscle bundles were isolated under a stereomicroscope for immunostaining.

### Nf-L analysis

Mouse blood was collected under isoflurane inhalation (1-3%) or during terminal procedures (4-5% of isoflurane), via submandibular sampling. Whole blood was allowed to clot at room temperature for 20–30 minutes, then centrifuged at 1500 g for 10–15 min at 4 °C. The resulting serum supernatant was isolated without disturbing the clot, aliquoted to avoid repeated freeze–thaw cycles, and stored at −80 °C until analysis. Serum Nf-L was measured by Serum NF-light ELISA assay (UmanDiagnostics 20-8002).

### qRT-qPCR

Total RNA was isolated using TRIzol reagent (Invitrogen). RNA was quantified by spectrophotometry using an absorbance of 260 nm, and the sample purity ratios were calculated (260/280 nm). Total RNA was extracted from different tissues for reverse transcription and h*SOD1* quantification using the 2^–ΔΔ^C_t_ method. The mouse *Hprt* gene was also measured as an endogenous reference. The probes (HS00533490_m1 SOD1-FAM; Mm01545399_m1 Hprt-Vic) are purchased from Thermo Scientific.

### Small RNA library construction and analysis

Total lumbar RNA was purified by 15% urea polyacrylamide gel electrophoresis, selecting for 18- to 30-nt-long RNAs. Ligation of the 3′ adaptor (5′-rApp TGG AAT TCT CGG GTG CCA AGG /ddC/−3′) was done using truncated, K227Q mutant T4 RNA Ligase 2 at 25 °C for ≥16 h. 5′ Adaptor (5′-GUU CAG AGU UCU ACA GUC CGA CGA UC-3′) was added using T4 RNA ligase (Ambion) at 25 °C for ≥2 h, followed by reverse transcription using avian myeloblastosis virus (AMV) reverse transcriptase and PCR using Q5 polymerase (NEB). An Illumina NextSeq550 was used for high-throughput, single-end 50-nt sequencing. The 3′ adaptors of small RNA libraries were removed using Flexbar 3.0.3. After trimming, only the reads with Phred scores at each position higher than 20 were kept for downstream analysis. Such reads were first aligned with Bowtie v.1.2.1.1 to miRNA hairpins supplemented with corresponding hairpin backbones of miR-33 and shRNA. Tailed reads were discovered with Tailor v.1.1. Differential analysis was done with DESeq v.3.5.

### RNA sequencing and bioinformatics

Strand-specific RNA-Seq libraries were prepared as previously described^98^. Paired-end RNA-Seq reads were first aligned to ribosomal RNA (BK000964.1) with Bowtie2^99^. Non-rRNA reads were subsequently used for gene quantification with Salmon v0.8.2^100^. Differential analysis was performed using DESeq v3.5^101^.

### Statistical analysis

Survival curve analyses were performed using the Log-rank (Mantel-Cox) test. The unpaired two-sided Student’s t test was performed to compare two groups, and a one-way analysis of variance (ANOVA) test, followed by the two-stage step-up (Benjamini, Krieger, and Yekutieli) test, was used to compare multiple groups beyond two. The Shapiro-Wilk test was conducted to ensure the data is normally distributed when n > 8 within a group. All data were analyzed by GraphPad Prism 10, and values are shown as mean ± standard deviation, unless otherwise specified. All data shown are biological replicates from distinct samples.

### Reporting summary

Further information on research design is available in the Nature Portfolio Reporting Summary linked to this article.

## Supplementary information

Supplementary Information

Reporting Summary

Transparent Peer Review file

## Source data

Source Data

## Data Availability

The raw NGS sequencing data of miRNA and mRNA in the mouse spinal cord have been deposited to the NCBI Sequence Read Archive (SRA) under the accession numbers SRA: PRJNA1306997 and PRJNA1306994. Other source data are provided in the Source Data file. Source data are provided with this paper.
