# Supplementary material for: Intravenous administration of an engineered AAV9-gene-silencing vector suppresses human SOD1 and extends survival in an ALS mouse model
Source: Nat Commun. 2026 Jun 25;17:5566. doi: 10.1038/s41467-026-74169-8 (PMC13303913; doi:10.1038/s41467-026-74169-8)
Supplement: Supplementary file 1 — Supplementary Information [file 41467_2026_74169_MOESM1_ESM.pdf]

Supplementary Table 1: Downregulation of h*SOD1* mRNA in the SOD1^{G93A} mice

		Remaining h*SOD1 mRNA levels by AAV9-amiR-SOD1 compared to the PBS group (%)*	
		Day 105	Day 216~277
CNS	Olfactory Bulb	76 ± 6 (P = 0.000531)	77 ± 11(P = 0.016363)
	Striatum	77 ± 11(P = 0.001443)	65 ± 9 (P = 0.000322)
	Cortex	99 ± 7 (P = 0.780701)	78 ± 12 (P = 0.009399)
	Hippocampus	81 ± 8 (P = 0.000232)	70 ± 19 (P = 0.021712)
	Cerebellum	83 ± 9 (P = 0.020090)	53 ± 8 (P = 0.003065)
	Brainstem	58 ± 12 (P = 0.000173)	57 ± 8 (P = 0.000519)
	Cervical	65 ± 12 (P = 0.002026)	59 ± 17 (P = 0.002878)
	Thoracic	56 ± 17 (P = 0.001262)	51 ± 11 (P =0.000342)
	Lumbar	57 ± 8 (P < 0.000001)	58 ± 10 (P = 0.000487)
	Sacral Coccygeal	71 ± 10 (P = 0.000075)	49 ± 8 (P = 0.000019)
Non-CNS	Tongue	65 ± 15 (P = 0.039244)	Tissue not collected
	Intercostal Muscles	40 ± 17 (P = 0.003926)	Tissue not collected
	Diaphragm	10 ± 3 (P < 0.000001)	26 ± 5 (P = 0.000012)
	Abdominal Muscles	26 ± 9 (P = 0.007742)	Tissue not collected
	Quadriceps	27 ± 12 (P = 0.000016)	24 ± 8 (P < 0.000001)
	Gastrocnemius	39 ± 18 (P = 0.000081)	39 ± 6 (P = 0.000001)
	Heart	11 ± 7 (P = 0.000672)	7 ± 1 *
	Liver	1 ± 1 (P < 0.000001)	66 ± 23 (P = 0.075537)
	Lung	72 ± 18 (P = 0.004780)	58 ± 7 (P = 0.017842)
	Bladder	77 ± 26 (P = 0.342687)	85 ± 1 *
	Intestine	90 ± 5 (P = 0.096414)	90 ± 19 (P = 0.431295)

*Statistics analysis was not done because the heart tissue was only harvested in two animals at the endpoint. A two-tailed Student's T-test was conducted between the PBS and vector-treated groups.

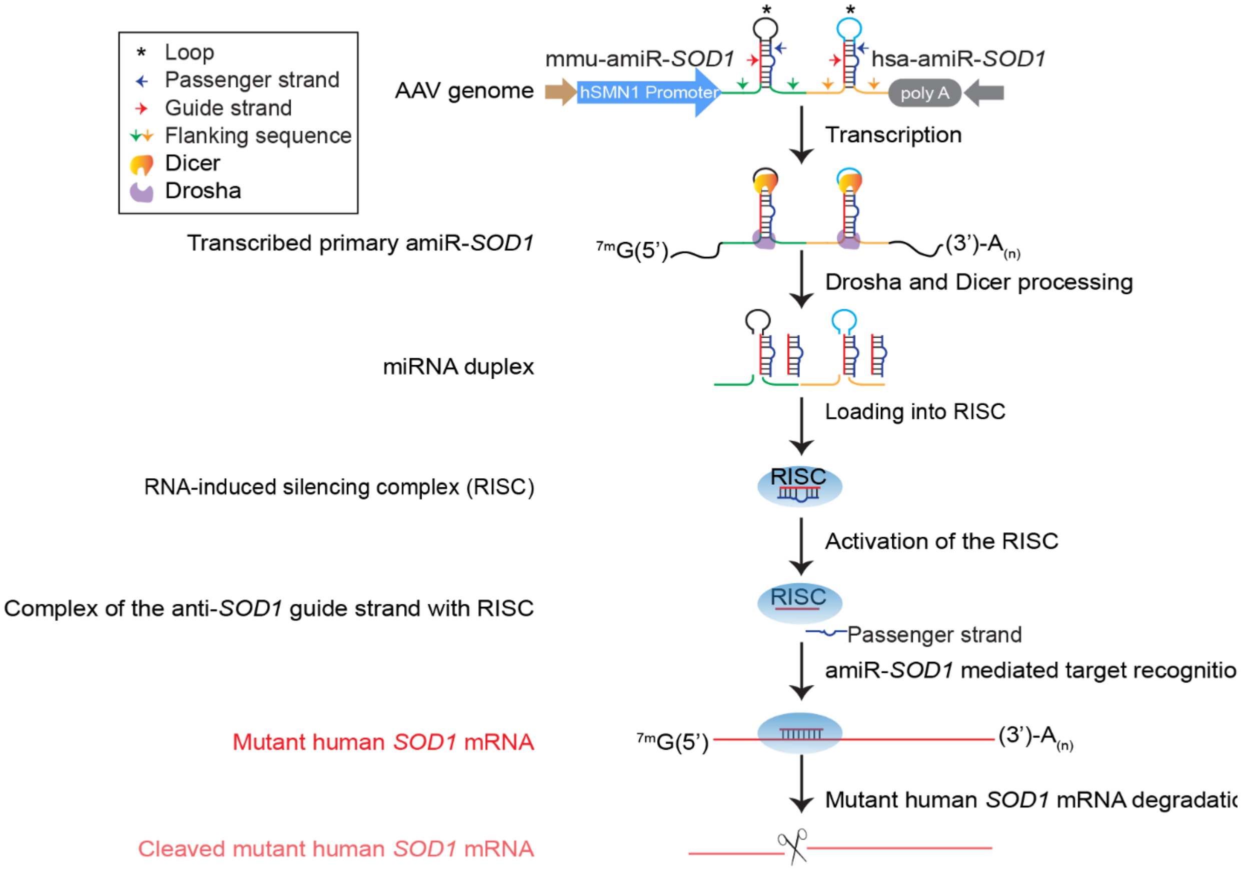

Supplementary Figure 1. Silencing of mutant human *SOD1* expression by amiR-*SOD1*. Under the h*SMN1* promoter, the same guide strand (red arrow) targeting the human *SOD1* gene was embedded in mouse and human *miR-33* scaffolds, respectively. These two scaffolds have identical stem-loop structures but differ in their flanking sequences and loops. Both amiR-*SOD1* were transcribed as pri-amiR and processed into miRNA duplex by Drosha and Dicer. The guide strand is preferentially loaded into the RISC to form a functional miRNA-induced silencing complex that suppresses mutant human *SOD1* expression.

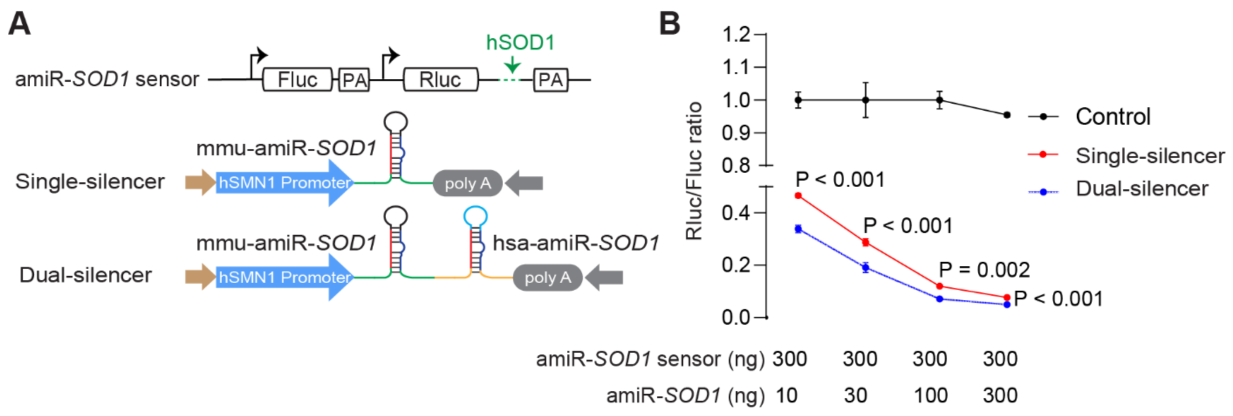

Supplementary Figure 2. Evaluation of amiR-*SOD1* RNAi potency in HEK293 cells.

(A) Schematics showing the amiR-*SOD1* sensor and the amiR-*SOD1* constructs. The amiR-*SOD1* sensor plasmid is a dual-luciferase reporter plasmid that carries the human *SOD1* coding sequence without the ATG start codon, downstream of the Renila luciferase (Rluc) reporter gene. Firefly luciferase (Fluc) is expressed as a reference. (B) amiRs silencing efficacies in HEK293 cells. The amiR-*SOD1* and its sensor plasmids at an indicated amount were co-transfected into HEK293 cells, and *Fluc* and *RLuc* levels were measured after 48 hours. Controls were co-transfected with the reporter plasmid and an EGFP plasmid. The Rluc /Fluc ratio was calculated to reflect gene-silencing efficacy. Comparison was done between the single-silencer (mmu-amiR-*SOD1*) and dual-silencer plasmids using multiple t-test. n = 3 wells per group. The dual silencer demonstrated a higher silencing capacity than the single silencer, particularly at low concentrations. Data is shown as mean ± SD.

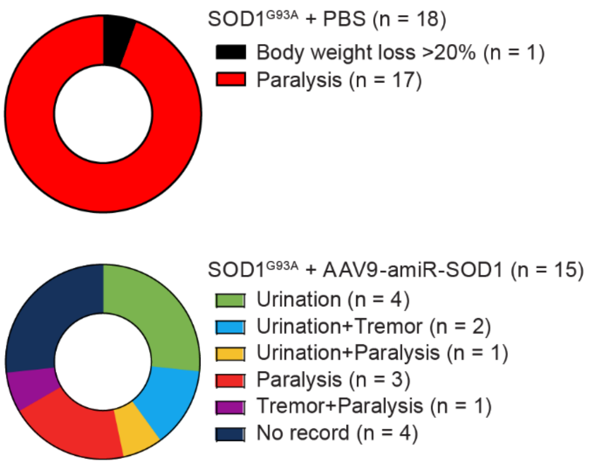

SupplemeSupplementaryntary Figure 3. Urinary and gastrointestinal symptoms in AAV9-amiR-*SOD1*-treated SOD1^{G93A} mice. ALS and non-ALS symptoms in SOD1^{G93A} mice at the end stage after treatment with PBS or AAV9-amiR-*SOD1*.

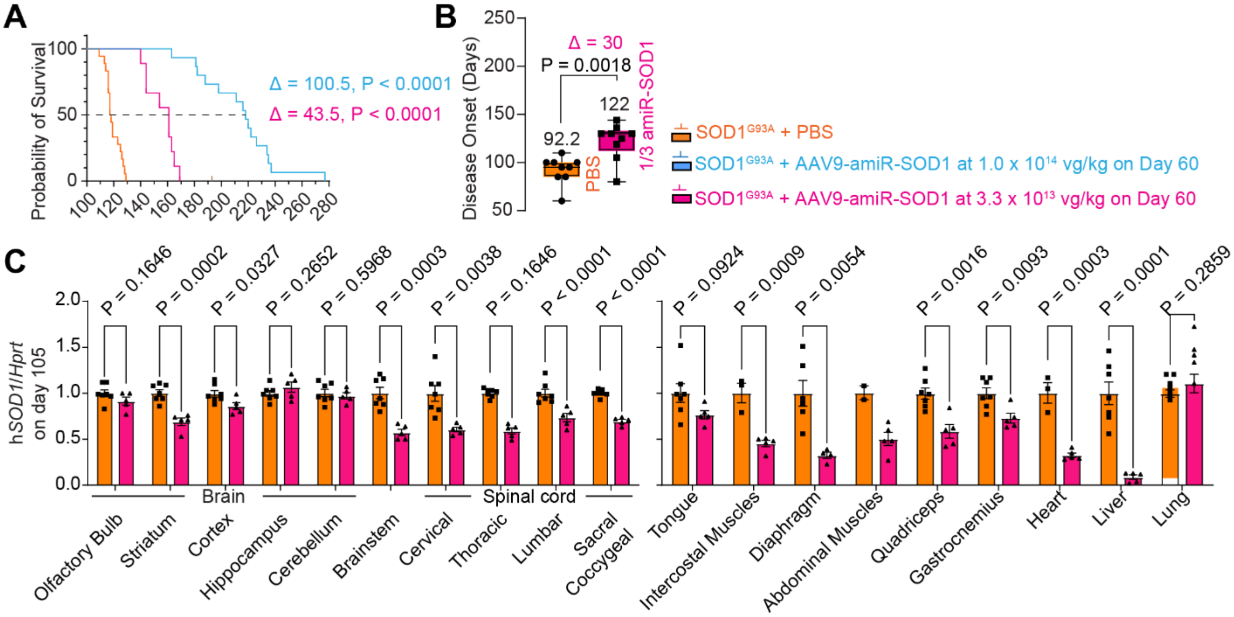

Supplementary Figure 4. Survival and disease onset modulation at reduced AAV9-amiR-*SOD1* dosing in SOD1^{G93A} mice. Kaplan-Meier survival plots (**A**) and disease onset (**B**) of SOD1^{G93A} mice (n = 8) receiving AAV9-amiR-*SOD1* at the low dose 3.3x10^{13} vg/kg on day 60 by IV injection. The PBS and the 10^{14} vg/kg dose are described elsewhere in this report. (**C**) h*SOD1* mRNA levels in the CNS and peripheral tissues at day 105 in SOD1^{G93A} mice treated with AAV9-amiR-*SOD1* at 3.3 x 10^{13} vg/kg or PBS on day 60 by IV injection (n = 5-7/group). Data is shown as mean ± SEM.

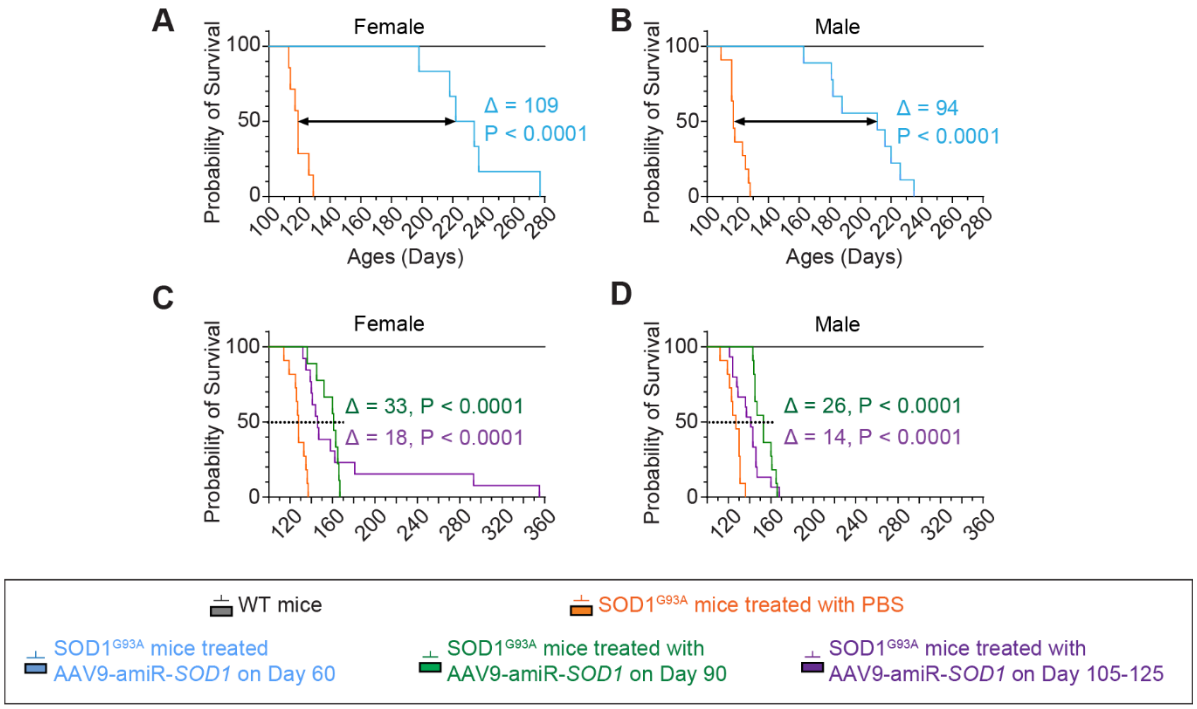

Supplementary Figure 5. Different gender responses to the vector treatment. (A) Kaplan-Meier survival plots of female SOD1^{G93A} mice receiving AAV9-amiR-*SOD1* (n = 8) or PBS (n = 7) on day 60. **(B)** Kaplan-Meier survival plots of male SOD1^{G93A} mice receiving AAV9-amiR-*SOD1* (n = 11) or PBS (n = 12) on day 60. **(C)** Kaplan-Meier survival plots of female *SOD1^{G93A}* mice receiving AAV9-amiR-*SOD1* on day 90 (n = 10) or day 105–125 (n = 13). **(D)** Kaplan-Meier survival plots of male *SOD1^{G93A}* mice receiving AAV9-amiR-*SOD1* on day 90 (n = 15) or day 105–125 (n = 16).

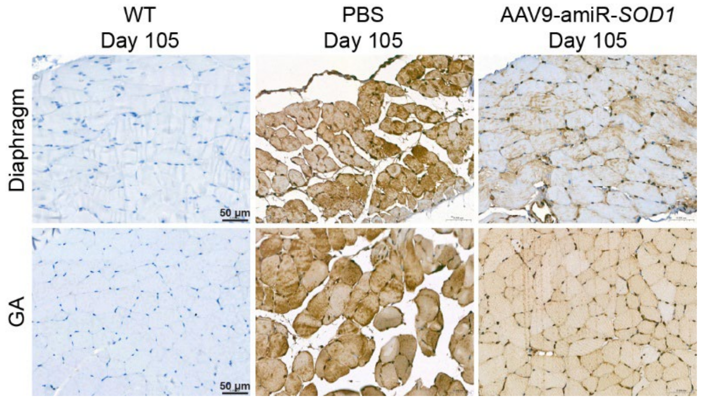

Supplementary Figure 6. AAV9-amiR-*SOD1* treatment lowered the hSOD1 expression in muscles. SOD1^{G93A} mice were treated on day 60, and the tissues were harvested on day 105. Muscle sections were stained with hSOD1 antibody and counterstained with hematoxylin.

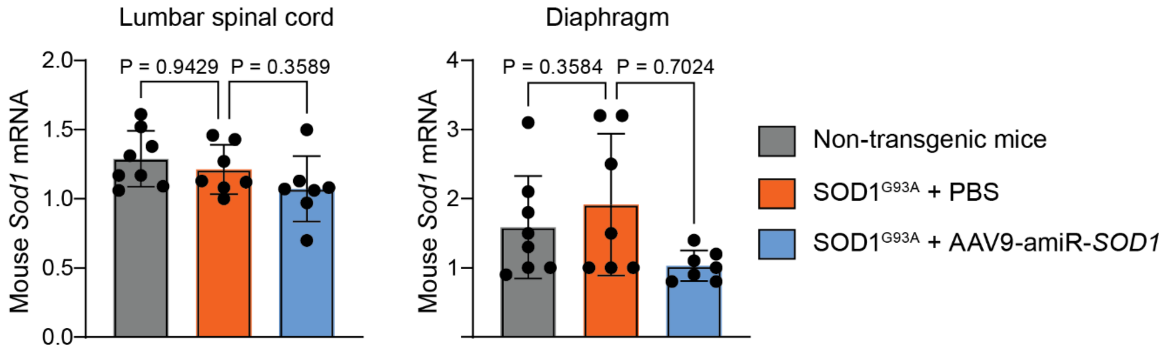

Supplementary Figure 7. AAV9-amiR-*SOD1* treatment did not change mouse *Sod1* mRNA levels in the lumbar spinal cord and diaphragm. The mice were treated on day 60 and analyzed on day 105. Age-matched WT were used for comparison. Mouse Sod1 expression was measured by qRT-PCR and normalized with endogenous *Hprt* level. Data is shown as mean ± SD.

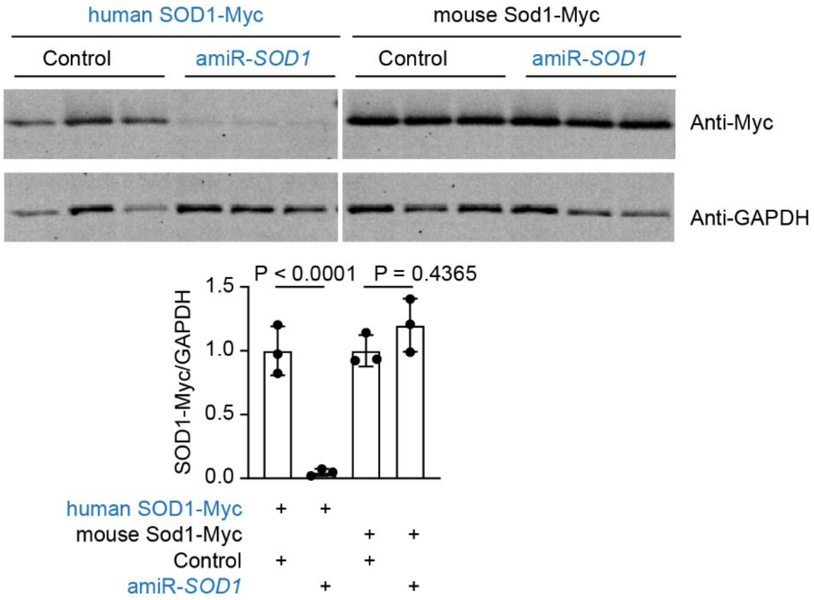

Supplementary Figure 8. AmiR-*SOD1* specifically downregulates human, but not mouse, SOD1 protein expression. Plasmids expressing human or mouse SOD1 fused with Myc-tag were co-transfected with the amiR-*SOD1* plasmids into HEK293 cells. After 48 hours, cell lysates were harvested for western blot analysis using the Myc antibody. Gapdh protein was used as a loading control. Data is shown as mean ± SD.

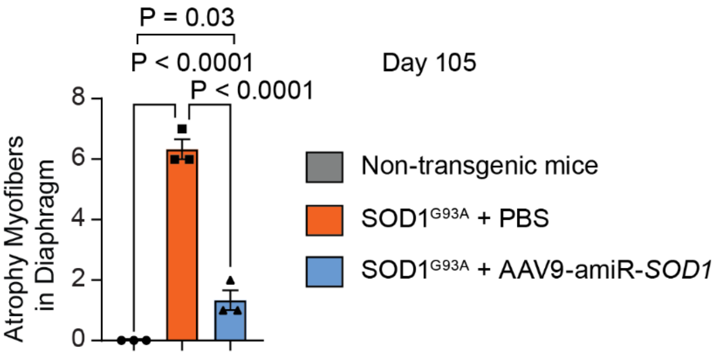

Supplementary Figure 9. Quantification of atrophic myofibers in SOD^{G93A} mice treated on day 60 and analyzed on day 105. The atrophic myofibers were counted in each visual frame at 40x magnification in three mice per group. Data is shown as mean ± SD.

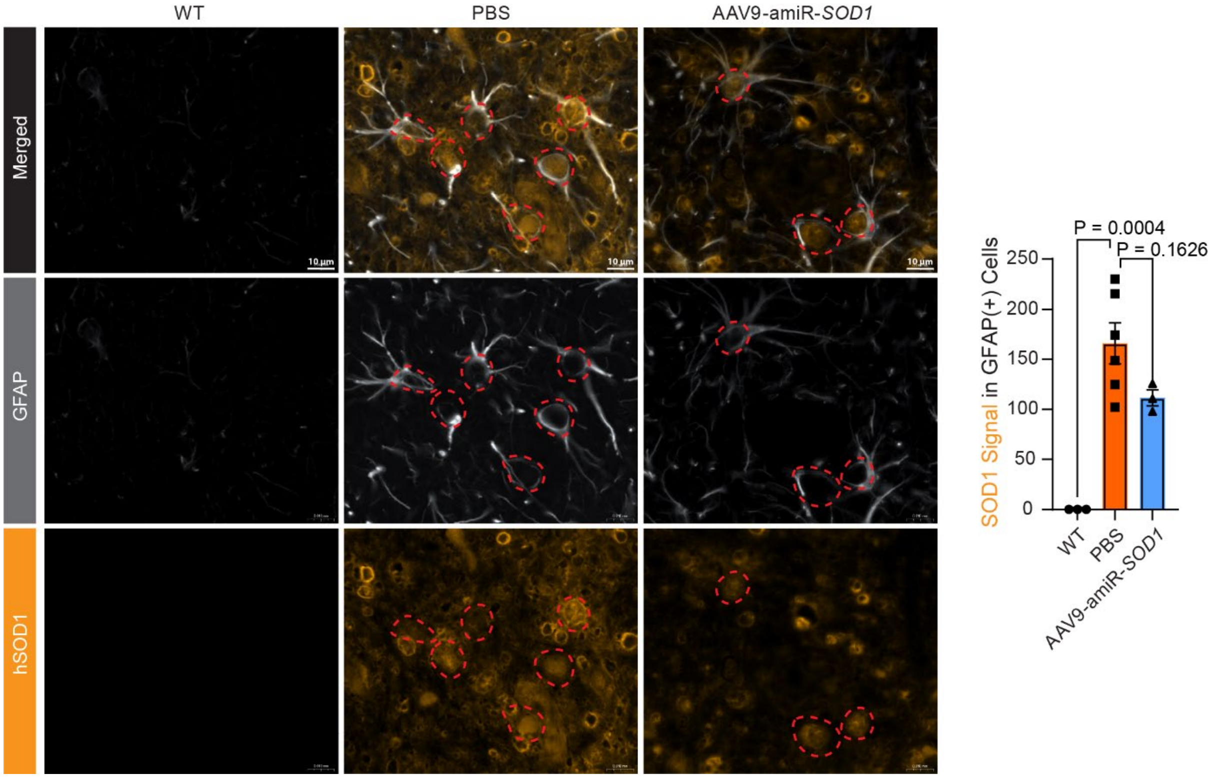

Supplementary Figure 10. AAV9-amiR-SOD1 did not knockdown hSOD1 in astrocytes. SOD1^{G93A} mice were treated on day 60 and analyzed on day 105. Lumbar spinal cord sections were co-stained for hSOD1 and Gfap. Age-matched WT mice were used for comparison. hSOD1 staining intensity was quantified in GFAP-positive cells, as shown on the right. Data is shown as mean ± SD.

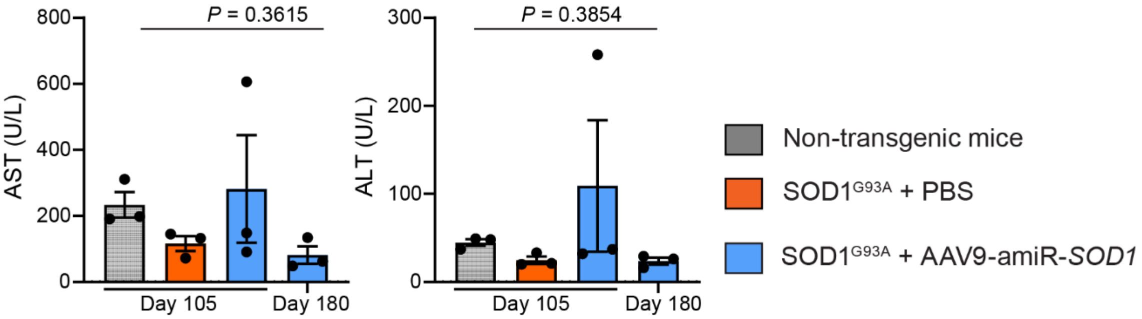

Supplementary Figure 11. AAV9-amiR-*SOD1* treatment did not alter liver function. The serum aspartate aminotransferase (AST) and alanine aminotransferase (ALT) were assayed to assess liver toxicity. Data is shown as mean ± SD.
