# Supplementary material for: Intravenous administration of an engineered AAV9-gene-silencing vector suppresses human SOD1 and extends survival in an ALS mouse model
Source: Nat Commun. 2026 Jun 25;17:5566. doi: 10.1038/s41467-026-74169-8 (PMC13303913; doi:10.1038/s41467-026-74169-8)
Supplement: Supplementary file 2 — Reporting Summary [file 41467_2026_74169_MOESM2_ESM.pdf]

nature portfolio

Corresponding author(s): Jun Xie

Last updated by author(s): Apr 25, 2026

Reporting Summary

Nature Portfolio wishes to improve the reproducibility of the work that we publish. This form provides structure for consistency and transparency in reporting. For further information on Nature Portfolio policies, see our Editorial Policies and the Editorial Policy Checklist.

Statistics

For all statistical analyses, confirm that the following items are present in the figure legend, table legend, main text, or Methods section.

n/a	Confirmed	
☐	☒	The exact sample size (*n*) for each experimental group/condition, given as a discrete number and unit of measurement
☐	☒	A statement on whether measurements were taken from distinct samples or whether the same sample was measured repeatedly
☐	☒	The statistical test(s) used AND whether they are one- or two-sided<br>*Only common tests should be described solely by name; describe more complex techniques in the Methods section.*
☐	☒	A description of all covariates tested
☐	☒	A description of any assumptions or corrections, such as tests of normality and adjustment for multiple comparisons
☐	☒	A full description of the statistical parameters including central tendency (e.g. means) or other basic estimates (e.g. regression coefficient) AND variation (e.g. standard deviation) or associated estimates of uncertainty (e.g. confidence intervals)
☐	☒	For null hypothesis testing, the test statistic (e.g. *F*, *t*, *r*) with confidence intervals, effect sizes, degrees of freedom and *P* value noted<br>*Give P values as exact values whenever suitable.*
☒	☐	For Bayesian analysis, information on the choice of priors and Markov chain Monte Carlo settings
☒	☐	For hierarchical and complex designs, identification of the appropriate level for tests and full reporting of outcomes
☒	☐	Estimates of effect sizes (e.g. Cohen's *d*, Pearson's *r*), indicating how they were calculated

Our web collection on statistics for biologists contains articles on many of the points above.

Software and code

Policy information about availability of computer code

Data collection	Leica SP-8 Laser Scanning Confocal Fluorescence, MoticEasyScan Infinity 60, 3D Histech Pannoramic MIDI II, Bowtie2, Illumina NextSeq550, Bowtie v.1.2.1.1,
Data analysis	GraphPad Prism 10, LAS X Office, fiji-windows-x64, DESeq v3.5, DESeq v.3.5,

For manuscripts utilizing custom algorithms or software that are central to the research but not yet described in published literature, software must be made available to editors and reviewers. We strongly encourage code deposition in a community repository (e.g. GitHub). See the Nature Portfolio guidelines for submitting code & software for further information.

Data

Policy information about availability of data

All manuscripts must include a data availability statement. This statement should provide the following information, where applicable:

- Accession codes, unique identifiers, or web links for publicly available datasets
- A description of any restrictions on data availability
- For clinical datasets or third party data, please ensure that the statement adheres to our policy

The RNAseq and miRseq data are deposited to NCBI (PRJNA1306997 and PRJNA1306994).

Research involving human participants, their data, or biological material

Policy information about studies with human participants or human data. See also policy information about sex, gender (identity/presentation), and sexual orientation and race, ethnicity and racism.

Reporting on sex and gender	This study did not involve human participants.
Reporting on race, ethnicity, or other socially relevant groupings	This study did not involve human participants.
Population characteristics	This study did not involve human participants.
Recruitment	This study did not involve human participants.
Ethics oversight	This study did not involve human participants.

Note that full information on the approval of the study protocol must also be provided in the manuscript.

Field-specific reporting

Please select the one below that is the best fit for your research. If you are not sure, read the appropriate sections before making your selection.

- [x] Life sciences
- [ ] Behavioural & social sciences
- [ ] Ecological, evolutionary & environmental sciences

For a reference copy of the document with all sections, see nature.com/documents/nr-reporting-summary-flat.pdf

Life sciences study design

All studies must disclose on these points even when the disclosure is negative.

Sample size	i) Fifteen SOD1G93A mice received an AAV injection on day 60, 25 SOD1G93A mice received an AAV injection on day 90 and 29 SOD1G93A mice received an AAV injection on day 105-125. These mice were used to collect survival and motor functional data.<br>ii) Seven SOD1G93A mice received an AAV injection on day 60 and sacrificed OMG day 105 for analysis.<br>iii) For human SOD1 expression quantification by qPT-PCR on day 105, n = 6-7/group. For all histology data a "n" of at least 3 animals was used. The "n" numbers were chosen based on previously published studies were gene therapy/silencing strategies were used in rodent SOD1-mutation-linked ALS models.
Data exclusions	During the study, animals that showed manifestations not related to ALS disease-linked behavioral phenotype, such severe fighting wounds were excluded from the study. These criteria were established based on our previous experience using this specific SOD1-G37R mouse model of ALS.
Replication	Highly significant treatment effect in all "in life" phase behavioral and "post-mortem" assays showed a high degree of reproducibility. The data from individual animals with animal's age in specific assays (spinal cord neuronal and muscle staining) are provided in corresponding figures.
Randomization	The animals were assigned to different experimental groups based on the litter and gender in a way that every experimental group had similar number of siblings males and females.
Blinding	All the animal experiments were conducted in a double-blind manner.

Reporting for specific materials, systems and methods

We require information from authors about some types of materials, experimental systems and methods used in many studies. Here, indicate whether each material, system or method listed is relevant to your study. If you are not sure if a list item applies to your research, read the appropriate section before selecting a response.

Materials & experimental systems

n/a	Involved in the study	
[ ]	[x]	Antibodies
[ ]	[x]	Eukaryotic cell lines
[x]	[ ]	Palaeontology and archaeology
[ ]	[x]	Animals and other organisms
[x]	[ ]	Clinical data
[x]	[ ]	Dual use research of concern
[x]	[ ]	Plants

Methods

n/a	Involved in the study	
[x]	[ ]	ChIP-seq
[x]	[ ]	Flow cytometry
[x]	[ ]	MRI-based neuroimaging

Antibodies

Antibodies used	For immunoflourescence staining the following antibodies were used: Human SOD1 (Mouse, Thermo Fisher, MA1-105, Clone 8B10, dilution 1:1000), ChAT (Goat, MilliporeSigma, ab144p, dilution 1:1000), GFAP (Mouse, Cell Signaling, 3670, dilution 1:200), IBA-1 (Rabbit, Cell Signaling, E4O4W, 17198, dilution 1:100), Synapsin (rabbit, Milipore-Sigma, anti-synapsin I, no. S193, dilution 1:50), Neurofilament (chicken, Milipore-Sigma, anti-neurofilament H, no. AB5539, dilution 1:500), Goat anti-Rabbit IgG (H+L) Highly Cross-Adsorbed Secondary Antibody, Alexa Fluor™ Plus 488 (Invitrogen, A32731, 1: 1000), Goat anti-Chicken IgY (H+L) Cross-Adsorbed Secondary Antibody, Alexa Fluor™ Plus 488 (Invitrogen, A32931, 1: 1000), Goat anti-Rat IgG (H+L) - Alexa Fluor Plus 488 (Invitrogen, A48262, 1:1000), Goat anti-Mouse IgG (H+L) - Alexa Fluor Plus 555 (Invitrogen, A32727, 1:1000), Goat anti-Rabbit IgG (H +L) - Alexa Fluor Plus 647(Invitrogen, A32733, 1:1000), Goat anti-Chicken IgY (H+L) - Cross-Adsorbed Secondary Antibody Dylight 755 (Invitrogen, SA5-10075, 1:1000)
Validation	The information on antibodies validation was taken from manufacturer's website(s) as follows:<br>MA1-105 antibody detects SOD1 in human and mouse samples and has been successfully used in western blot, immunohistochemistry, immunocytochemistry and immunofluorescence applications.<br>ChAT antibody detects choline acetyltransferase in human, opossum, guinea pig, rat, mouse, zebrafish, avian, monkey, chicken and has been successfully used in western blot, immunohistochemistry, immunocytochemistry and immunofluorescence applications.<br>GFAP (GA5) Mouse mAb detects endogenous levels of total GFAP protein in human, mouse, rat and has been successfully used in western blot, immunohistochemistry, flow cytometry and immunofluorescence applications.<br>Iba1/AIF-1 (E4O4W) XP® Rabbit mAb recognizes endogenous levels of total Iba1/AIF-1 protein in human, mouse, rat, hamster, monkey and has been successfully used in western blot, simple western™, immunoprecipitation, IHC leica bond, immunohistochemistry (paraffin), immunofluorescence (frozen), immunofluorescence (immunocytochemistry), flow cytometry (fixed/permeabilized).<br>Synapsin I antibody is be used to localize and detect synapsin I (synapsins Ia and Ib are collectively referred to as synapsin I) in nerve terminals in rat, mouse, human, bovine. The SYN1 gene is mapped to human chromosome Xp11.3-p11.23. This antibody has been successfully used in western blotting, immunohistochemistry, immunoblotting, immunoprecipitation, ELISA.<br>Anti-Neurofilament H antibody recognizes neurofilament-heavy (NF-H). The antibody stains sharply defined cytoplasmic filaments in neuronal cell bodies and especially axons in rat, pig, bovine, feline, human, mouse and has been successfully used in western blot, immunohistochemistry, immunocytochemistry and immunofluorescence applications.

Eukaryotic cell lines

Policy information about cell lines and Sex and Gender in Research

Cell line source(s)	*State the source of each cell line used and the sex of all primary cell lines and cells derived from human participants or vertebrate models.*
Authentication	*Describe the authentication procedures for each cell line used OR declare that none of the cell lines used were authenticated.*
Mycoplasma contamination	*Confirm that all cell lines tested negative for mycoplasma contamination OR describe the results of the testing for mycoplasma contamination OR declare that the cell lines were not tested for mycoplasma contamination.*
Commonly misidentified lines<br>(See ICLAC register)	*Name any commonly misidentified cell lines used in the study and provide a rationale for their use.*

Animals and other research organisms

Policy information about studies involving animals; ARRIVE guidelines recommended for reporting animal research, and Sex and Gender in Research

Laboratory animals	Parental male B6SJL-Tg (SOD1*G93A)1Gur/J mice (JAX: 002726) and female non-transgenic mice (JAX: 100012) at 30 days of age were obtained from Jackson Laboratory. Parental mice were mated in the UMass Chan Medical School Animal Facility. Experimental mice transgene copy numbers were monitored by ddPCR using the primer/probe set provided by Jackson Laboratory. The animal experiments were approved by the Institutional Animal Care and Use Committee (Protocol #: PROTO202000037 and IPROTO202400000060) of UMass Chan Medical School
Wild animals	This study did not involve wild animals.
Reporting on sex	Each experimental group had similar number of siblings males and females.
Field-collected samples	This study did not involve samples collected from the field.
Ethics oversight	All experiments were performed in accordance with the NIH Guidelines and approved by the Institutional Animal Care and Use Committee (IACUC) of UMass Chan Medical School.

Note that full information on the approval of the study protocol must also be provided in the manuscript.

Plants

Seed stocks	*Report on the source of all seed stocks or other plant material used. If applicable, state the seed stock centre and catalogue number. If plant specimens were collected from the field, describe the collection location, date and sampling procedures.*
Novel plant genotypes	*Describe the methods by which all novel plant genotypes were produced. This includes those generated by transgenic approaches, gene editing, chemical/radiation-based mutagenesis and hybridization. For transgenic lines, describe the transformation method, the number of independent lines analyzed and the generation upon which experiments were performed. For gene-edited lines, describe the editor used, the endogenous sequence targeted for editing, the targeting guide RNA sequence (if applicable) and how the editor was applied.*
Authentication	*Describe any authentication procedures for each seed stock used or novel genotype generated. Describe any experiments used to assess the effect of a mutation and, where applicable, how potential secondary effects (e.g. second site T-DNA insertions, mosiacism, off-target gene editing) were examined.*
