# Supplementary material for: Intravenous administration of an engineered AAV9-gene-silencing vector suppresses human SOD1 and extends survival in an ALS mouse model
Source: Nat Commun. 2026 Jun 25;17:5566. doi: 10.1038/s41467-026-74169-8 (PMC13303913; doi:10.1038/s41467-026-74169-8)
Supplement: Supplementary file 3 — Transparent Peer Review file [file 41467_2026_74169_MOESM3_ESM.pdf]

nature portfolio

Peer Review File

Intravenous administration of an engineered AAV9-gene-silencing vector suppresses human SOD1 and extends survival in an ALS mouse model

Corresponding Author: Professor Jun Xie

This file contains all reviewer reports in order by version, followed by all author rebuttals in order by version.

Version 0:

Reviewer comments:

Reviewer #1

(Remarks to the Author)
Wan et report on experiments that explore the therapeutic use of a new recombinant AAV9 construct to silence the expression of human mutant SOD1 in transgenic mouse model of ALS. The authors use a single iv dose of AA9-amiRNA-SOD1 and examine disease onset, survivability, and pathology in skeletal muscle and spinal cord. The design and construction of the new AAV9 is thoughtful and impressive. The results shown are very impressive regarding the efficacy of human SOD1 mRNA expression knockdown, lifespan extension, protection of neurological function, and rescue from the skeletal muscle and spinal cord pathology.

The manuscript has some weaknesses in the experimental design and results presentation that they could consider to improve the scientific reporting.
1. It is very unfortunate that the design of the experiments with the appropriate controls was not considered fully. They used PBS injected mice as the controls. The design would have been better if they used single dose iv injection of an AA9 encoding GFP or the miR-33 cassette without the amiR-SOD1 sequence or a scrambled sequence that could not target human SOD1. Systemic delivery of AAV9 can in itself have many biological effects, including immunomodulation. The virus administration needs to be controlled for.
2. The figure legends were not in the manuscript.
3. Though the qRTPCR results show extraordinary knockdown of human SOD1 expression (Figure 2), it was incomplete. The authors should show how much residual human SOD1 protein is expressed in skeletal muscle and spinal cord.
4. Data should be shown that endogenous mouse SOD1 is not affected at mRNA and protein levels in the presence of AAV9-amiRNA-SOD1.
5. Generally, the histology shown in the figures is suboptimal, particularly the spinal cord staining in Figure 3C and the immunohistochemistry in Figure 5.
6. The authors identify a region in the medulla called the ventral respiratory group (VRG). This VRG is very important and needs to be shown at lower magnification to give the reader perspective on the location of the VRG in the medulla. Based on figure 3c it looks to be very close to the ventrolateral surface of the medulla. Please clarify the location of the VGR.
7. In figure 3c (right column) it is not clear what the black arrows are pointing to. Are these blood vessels or swollen astrocytes? Structure identification needs to be done.
8. In figure 3d the NMJs for the PBS treatment mouse are not shown en face. They are shown from the side. It is not a good comparison with the other treatment groups.
9. In figure 5a the hSOD1 image for the PBS group is out of focus. Please remove the outlines of the motor neurons as these obscure the cell visualization.
10. Figure 5c requires some follow-up structure identification for the open profiles regarding whether they are blood vessels of swollen astrocytes.
11. As an additional control for AAV9 safety the WT mice could be treated with the AAV9
12. The introduction length too long and should be shortened by ¼ page.
13. While the choice of mouse model for this experimental design is appropriate it is important for the authors to acknowledge that therapeutic testing with this model has not robustly translated to clinical efficacy.

Reviewer #3

(Remarks to the Author)
The authors describe an AAV9-based gene therapy delivering dual amiR-SOD1 silencers under the hSMN1 promoter in the SOD1-G93A ALS model. The therapy delays onset, extends survival by over 100 days, preserves neuromuscular function, and reduces serum neurofilament light chain (Nf-L) levels by up to 90%. Given that Nf-L is an established biomarker and the regulatory basis for tofersen approval, these findings are of significant translational interest. The study's efficacy surpasses prior SOD1-directed interventions. However, limitations in mechanistic explanation, cortical/astrocytic silencing, and adverse disease-specific side effects warrant deeper consideration before acceptance in Nature Communications. Sample sizes are good

1. The authors should have a more direct comparison to Tofersen to better exemplify biomarker translation. The inclusion of serum Nf-L analysis is a major strength. Given that Nf-L reduction was central to tofersen's regulatory approval, the authors should more explicitly highlight this translational bridge. A comparative discussion of how AAV-amiR-SOD1's Nf-L lowering relates to tofersen's magnitude and kinetics would strengthen the manuscript's clinical relevance.

2. There is insufficient description and analysis of the mechanism of efficacy. While the construct design is innovative, the extraordinary therapeutic benefit observed requires mechanistic clarity. Specifically, is the magnitude of Nf-L lowering proportional to SOD1 knockdown, and why is efficacy robust despite incomplete cortical and astrocytic silencing? More detail on vector biodistribution, promoter activity, and cell type–specific silencing is needed.

3. There are major safety and off-target concerns that could greatly impede clinical translation. The observed urinary and gastrointestinal complications remain concerning. Even if disease-specific, further exploration is necessary to rule out vector-driven or off-target effects. Additionally, toxicology data (e.g., liver enzymes, immune profiling) are essential given the high systemic dose used.

4. As the authors are quite aware, while the SOD1-G93A model is important and does represent a meaningful portion of ALS cases, it still does not represent the majority of cases. All efficacy data are from SOD1-G93A mice. While this is the standard and most widely ALS model, extension to additional ALS-relevant models (e.g., SOD1-G37R, TDP-43, or FUS) would improve confidence in broader applicability.

5. The authors use t-test and ANOVA with post-hoc correction for some key analyses. The authors need to examine the data with Shapiro-Wilk or Kolmogorov-Smirnov test, etc. to ensure it meets the sufficiently normal distribution that is assumed by these methods. While it would be expected this would be true, this needs to done for completeness and appropriate rigor.

MINOR

1. Could the authors please elaborate on sex-specific differences in treatment response?

2. Supplementary figures should more clearly display individual animal trajectories in addition to group averages to allow better assessment of variability.

3. AAV9 was delivered at 1×10^14 v/kg. Even though it has been used in pediatric populations, it still a relatively high systemic dose. The authors could better justify this choice (e.g., prior dose-response pilot data) and discussion of translational feasibility. (This reviewer does note that lines 291-405 briefly address this concern. However, more discussion and justification would enhance the study credibility and translatability.)

4. If possible, more comparisons to harmonized baselines would help provide additional support.

Version 1:

Reviewer comments:

Reviewer #3

(Remarks to the Author)
The reviewers have made excellent efforts to address all reviewer critiques. They have extended the statistical analysis, made important experimental and mechanistic clarifications, and refined wording in the interpretations / Discussion to avoid excessive mechanistic or translational claims. The edits have improved analytical rigor, transparency, and appropriately matched evidence to claims. The paper is much improved in quality and readability.

Open Access This Peer Review File is licensed under a Creative Commons Attribution 4.0 International License, which permits use, sharing, adaptation, distribution and reproduction in any medium or format, as long as you give appropriate credit to the original author(s) and the source, provide a link to the Creative Commons license, and indicate if changes were made.
In cases where reviewers are anonymous, credit should be given to 'Anonymous Referee' and the source.
The images or other third party material in this Peer Review File are included in the article's Creative Commons license, unless indicated otherwise in a credit line to the material. If material is not included in the article's Creative Commons license and your intended use is not permitted by statutory regulation or exceeds the permitted use, you will need to obtain permission directly from the copyright holder.
To view a copy of this license, visit https://creativecommons.org/licenses/by/4.0/

Response to the reviewers:

Reviewer #1 (Remarks to the Author):

Wan et report on experiments that explore the therapeutic use of a new recombinant AAV9 construct to silence the expression of human mutant SOD1 in transgenic mouse model of ALS. The authors use a single iv dose of AA9-amiRNA-SOD1 and examine disease onset, survivability, and pathology in skeletal muscle and spinal cord. The design and construction of the new AAV9 is thoughtful and impressive. The results shown are very impressive in terms of the efficacy of human SOD1 mRNA expression knockdown, lifespan extension, protection of neurological function, and rescue from skeletal muscle and spinal cord pathology.

We appreciate the reviewer's encouraging comments.

The manuscript has some weaknesses in the experimental design and results presentation that they could consider to improve the scientific reporting.

1. It is very unfortunate that the design of the experiments with the appropriate controls was not considered fully. They used PBS injected mice as the controls. The design would have been better if they used single dose iv injection of an AA9 encoding GFP or the miR-33 cassette without the amiR-SOD1 sequence or a scrambled sequence that could not target human SOD1. Systemic delivery of AAV9 can in itself have many biological effects, including immunomodulation. The virus administration needs to be controlled for.

AAV-mediated in vivo gene transfer has been evaluated in mouse models of many CNS diseases, including ALS. PBS or AAV-GFP vector was injected into the same SOD1 mice via IV (PMID: 24008656, 26710998) or other routes (PMID: 37928442, 30195799) as controls to evaluate therapeutic outcomes. None of these treatments showed any therapeutic benefits. While it is theoretically possible, in practice, it is extremely unlikely because there is no evidence from the abundant literature reporting AAV9 transduction or amiR expression benefits in ALS. Instead, what we have observed is a strong knockdown of SOD1 but little evidence for the commonly observed side effects, e.g., inflammation, perturbed endogenous miRNA levels, and large alterations of gene expression profiles.

2. The figure legends were not in the manuscript.

We apologize for this oversight and have ensured that the figure legend is included in this submission.

3. Though the qRTPCR results show extraordinary knockdown of human SOD1 expression (Figure 2), it was incomplete. The authors should show how much residual human SOD1 protein is expressed in skeletal muscle and spinal cord.

We agree with this point. The new data on residual human SOD1 protein levels in muscle is provided in Supplementary Figure 6. The data on residual human SOD1 protein levels in the spinal cord was shown in Fig. 5A-B and Fig. 6A-B. We have tried to improve the clarity of the descriptions in this revised manuscript (lines 162-163; 248-252).

4. Data should be shown that endogenous mouse SOD1 is not affected at mRNA and protein levels in the presence of AAV9-amiRNA-SOD1.

We agree with this point. We have added new data showing that the mouse Sod1 protein level was not affected by amiR-*SOD1,* as shown in Supplementary Figure 8 (new). The mouse Sod1 mRNA data was shown as Supplementary Figure 7.

5. Generally, the histology shown in the figures is suboptimal, particularly the spinal cord staining in Figure 3C and the immunohistochemistry in Figure 5.

We have updated Figure 3C and Figure 5 to improve the quality.

6. The authors identify a region in the medulla called the ventral respiratory group (VRG). This VRG is very important and needs to be shown at lower magnification to give the reader perspective on the location of the

VRG in the medulla. Based on figure 3c it looks to be very close to the ventrolateral surface of the medulla. Please clarify the location of the VRG.

We agree with this point and have added demarcations surrounding the VRG region in the revised Fig. 3C.

7. In figure 3c (right column) it is not clear what the black arrows are pointing to. Are these blood vessels or swollen astrocytes? Structure identification needs to be done.

We have added clarifications about this and other symbols in the figures in this revision (lines 210-212). We thank the reviewer for pointing out this unclarity.

8. In figure 3d the NMJs for the PBS treatment mouse are not shown en face. They are shown from the side. It is not a good comparison with the other treatment groups.

The figure 3D is revised following the reviewer's suggestion.

9. In figure 5a the hSOD1 image for the PBS group is out of focus. Please remove the outlines of the motor neurons as these obscure the cell visualization.

We have removed the outlines of the motor neurons in the WT and PBS groups. We also reexamined our images and concluded that the appearance of fuzzy hSOD1 signal in the PBS group is due to the very high abundance of hSOD1 protein. The same section was scanned with the same settings for ChAT and GFAP, and both signals are clear.

10. Figure 5c requires some follow-up structure identification for the open profiles regarding whether they are blood vessels of swollen astrocytes.

The vacuoles are well-established structures in this mouse model and have been shown to originate from mitochondria (PMID: 7605627, 9547233, 16642310,12390515). These references are also cited in the manuscript text to help interested readers follow the structural identification.

11. As an additional control for AAV9 safety the WT mice could be treated with the AAV9

We understand this concern and plan to include this control in a formal toxicity study in WT mice as part of an IND. However, we would like to point out that ample investigation has been conducted on this new vector design. In our previous study, we demonstrated that the miR-33 scaffold is a substantial improvement over previously reported shRNA/miRNA scaffolds. Embedding the siRNA into the miR-33 scaffold efficiently silenced the target, reduced off-target activity by 10-fold, and produced fewer truncated genomes compared to conventional shRNA design (PMID: 31843447). By using the SMN1 promoter, we further improved the safety of AAV9 gene therapy for the treatment of SMA (PMID: 38413838). In the present study, we profiled miRNAs and transcriptome and found that deviations from the wild type profiles caused by the expression of mutant SOD1 were diminished by the vector-treatment (Fig. 7). Additionally, our liver function gauge, the ALT (alanine aminotransferase) and AST (aspartate aminotransferase) levels, were unchanged following the AAV9-amiR-SOD1 treatment (Supplementary Figure 11). This is strong evidence supporting the safety of this vector and the further development of its clinical application.

12. The introduction length too long and should be shortened by ¼ page.

We have shortened the introduction as the reviewer suggested.

13. While the choice of mouse model for this experimental design is appropriate it is important for the authors to acknowledge that therapeutic testing with this model has not robustly translated to clinical efficacy.

We appreciate this suggestion and have added discussion about this point in the manuscript (lines 431-439).

Reviewer #3 (Remarks to the Author):

The authors describe an AAV9-based gene therapy delivering dual amiR-SOD1 silencers under the hSMN1

promoter in the SOD1-G93A ALS model. The therapy delays onset, extends survival by over 100 days, preserves neuromuscular function, and reduces serum neurofilament light chain (Nf-L) levels by up to 90%. Given that Nf-L is an established biomarker and the regulatory basis for tofersen approval, these findings are of significant translational interest. The study's efficacy surpasses prior SOD1-directed interventions. However, limitations in mechanistic explanation, cortical/astrocytic silencing, and adverse disease-specific side effects warrant deeper consideration before acceptance in Nature Communications. Sample sizes are good.

We appreciate this reviewer's encouraging comments.

1. The authors should have a more direct comparison to Tofersen to better exemplify biomarker translation. The inclusion of serum Nf-L analysis is a major strength. Given that Nf-L reduction was central to tofersen's regulatory approval, the authors should more explicitly highlight this translational bridge. A comparative discussion of how AAV-amiR-SOD1's Nf-L lowering relates to tofersen's magnitude and kinetics would strengthen the manuscript's clinical relevance.

We thank the reviewer for the suggestion. We have incorporated this suggestion in the Discussion section (lines 400-406; 407-420).

2. There is insufficient description and analysis of the mechanism of efficacy. While the construct design is innovative, the extraordinary therapeutic benefit observed requires mechanistic clarity. Specifically, is the magnitude of Nf-L lowering proportional to SOD1 knockdown, and why is efficacy robust despite incomplete cortical and astrocytic silencing? More detail on vector biodistribution, promoter activity, and cell type–specific silencing is needed.

We really appreciate these points and have made numerous revisions throughout this manuscript to improve clarity. We generated a diagram illustrating the processing of the dual-amiR, shown in Supplementary Figure 1. We discussed the contributing factors for this drastically improved therapeutic benefit (lines 347-365). We hope that the revisions have answered all the questions raised.

3. There are major safety and off-target concerns that could greatly impede clinical translation. The observed urinary and gastrointestinal complications remain concerning. Even if disease-specific, further exploration is necessary to rule out vector-driven or off-target effects. Additionally, toxicology data (e.g., liver enzymes, immune profiling) are essential given the high systemic dose used.

These are all valid and excellent points. Clinical application of gene therapy remains challenging, and we do not claim that all problems are solved. However, we believe that our work has made significant advances. GI and urinary symptoms have been observed in mouse models and ALS patients, and in previous AAV-delivered gene silencing experiments (PMID: 26891182, 7647793, 21989245). Therefore, it is unlikely that the vector treatment causes these symptoms. Rather, they likely resulted from insufficient therapy. For example, we detected no mutant hSOD1 knockdown in the bladder and intestine. These problems remain to be solved in future experiments.

We have addressed the liver toxicity issue in our response to reviewer 1's point 11, providing new data in Supplementary Figure 11.

4. As the authors are quite aware, while the SOD1-G93A model is important and does represent a meaningful portion of ALS cases, it still does not represent the majority of cases. All efficacy data are from SOD1-G93A mice. While this is the standard and most widely ALS model, extension to additional ALS-relevant models (e.g., SOD1-G37R, TDP-43, or FUS) would improve confidence in broader applicability.

This is another great point with which we agree. While we eye on a broadly applicable ALS treatment, we must make progress step by step. The key is to make persistent effort to push the therapeutic frontier and that's exactly what we and the neurodegenerative disease field will do. To address the reviewer's point, we have added discussions on the advances and limitations of our current results. We point out that our results are applicable to the SOD1-ALS cases but not to the sporadic cases in the discussion section. We also point out that the advances that we have achieved illustrate the therapeutic potential for other diseases caused by dominant gain of toxicity type of gene mutations.

5. The authors use t-test and ANOVA with post-hoc correction for some key analyses. The authors need to examine the data with Shapiro-Wilk or Kolmogorov-Smirnov test, etc. to ensure it meets the sufficiently normal distribution that is assumed by these methods. While it would be expected this would be true, this needs to done for completeness and appropriate rigor.

We have conducted the Shapiro-Wilk test on the disease onset data in Figure 1C (AAV9-amiR-SOD1, P = 0.85; PBS, P = 0.10) and Figure 1F (AAV9-amiR-*SOD1, P* = 0.16*;* PBS, *P* = 0.19). Both data sets were normally distributed. We also updated the Method to include this information (lines 607-609).

MINOR

1. Could the authors please elaborate on sex-specific differences in treatment response?

In general, the female mice responded better to the treatment. We summarized the survival data on gender differences in new Supplementary Figure 5.

.

2. Supplementary figures should more clearly display individual animal trajectories in addition to group averages to allow better assessment of variability.

We have examined all the supplementary figures and made necessary revisions to display individual animals wherever applicable.

3. AAV9 was delivered at 1×10^14 v/kg. Even though it has been used in pediatric populations, it still a relatively high systemic dose. The authors could better justify this choice (e.g., prior dose-response pilot data) and discussion of translational feasibility. (This reviewer does note that lines xxx briefly address this concern. However, more discussion and justification would enhance the study credibility and translatability.)

The dose at 1×10^14 vg/kg was chosen because 1.1×10^14 vg/kg by IV is used to treat SMA patients (lines 114-115). Currently, a Phase III clinical trial using AAV8-micro-Dystropin at a dose of 2×10^14 vg/kg by intravenous injection for the treatment of Duchenne Muscular Dystrophy has resulted in minimal adverse events (https://regenxbiodmdtrials.com/), even in a 33-kg patient who was well tolerated despite a heavy immunosuppressive regimen (personal communication). It is possible that ALS patients weighing ~70 kg can be treated with 1.0×10^14 vg/kg by IV. However, we did experiments with a lower dose (3.3×10^13 vg/kg at day 60) and it also demonstrated therapeutic benefit. We have included this data in Supplementary Figure 4 and described the result in the text (lines 130-133). We also discussed the translational feasibility in lines 431-457.

4. If possible, more comparisons to harmonized baselines would help provide additional support.

The median survival of PBS-treated $SOD1^{G93A}$ mice is 118 ± 6 and 128 ± 7 days in Figures 1B and 1E, respectively. In both cases, the mice assigned to the PBS and AAV9-amiR-SOD1 groups have similar hSOD1 transgene copy numbers. We included this information in the Methods (lines 486-487).
